# Galla Chinensis Polyphenol-Loaded Hemostatic Granules for Rapid Hemostasis, Antibacterial Action, and Wound Healing Promotion

**DOI:** 10.3390/jfb17060260

**Published:** 2026-05-25

**Authors:** Ruoxue Guo, Zihan Wu, Zirui He, Changsheng Liu, Yuan Yuan

**Affiliations:** 1 Key Laboratory for Ultrafine Materials of Ministry of Education, School of Materials Science and Engineering, East China University of Science and Technology, Shanghai 200237, China; y30230869@mail.ecust.edu.cn (R.G.); liucs@ecust.edu.cn (C.L.); 2 Engineering Research Center for Biomedical Materials of the Ministry of Education, East China University of Science and Technology, Shanghai 200237, China; 3 Shanghai Rebone Biomaterials Co., Ltd., Shanghai 201707, China; wuzihan@rebone.com

**Keywords:** hemostatic granules, Galla chinensis polyphenols, antibacterial, anti-inflammation, wound healing

## Abstract

Uncontrolled bleeding, coagulation disorders, and infection-related complications still present substantial challenges in emergency medicine and trauma care. Developing multifunctional hemostatic materials represent an effective strategy for addressing clinical hemostasis problems. In this study, Galla chinensis polyphenols, the effective extract of Galla chinensis, were loaded onto calcium alginate-mesoporous silica granules (CMS-GC). The CMS granules were prepared by in situ liquid-phase technology and GC was loaded by impregnation methods. In vitro and in vivo studies showed that CMS-GC not only activate the endogenous coagulation pathway via GC, but also the multi-level interconnected pores of CMS granules can promote the cross-linking of GC with plasma proteins and formation of a three-dimensional network structure, which further enhances the coagulation effect and shortens the blood clotting time to less than 80 s. In rat liver and femoral artery hemorrhage models, CMS-GC significantly shortened hemostasis time and reduced blood loss, demonstrating superior hemostatic performance. Moreover, within the moist environment sustained by alginate, GC mitigates inflammatory responses via its antibacterial and free-radical clearance properties, and synergistically facilitates wound healing. This CMS-GC multifunctional granule provides an efficient new strategy for traumatic bleeding and subsequent repair.

## 1. Introduction

Uncontrolled post-traumatic bleeding, coagulation dysfunction, and complications resulting from infections still pose substantial clinical challenges in emergency medicine and trauma care nowadays [1,2]. Over 40% of fatalities following severe trauma are due to a bleeding-induced pathophysiological cascade, which induces coagulation dysfunction, sustains inflammation, and promotes infectious complications, ultimately leading to impaired tissue repair and multiorgan dysfunction or failure [3,4]. Therefore, the development of a multifunctional integrated strategy capable of rapidly achieving hemostasis in the early stage of trauma and establishing an antibacterial and antioxidant environment to promote wound healing is greatly anticipated in trauma hemostasis and treatment [5,6].

Existing hemostatic materials, such as amorphous aluminosilicates and alginates, primarily depend on physical blood clotting and electrostatic interactions to reduce bleeding volume [7,8,9,10]. These processes often require hemostatic materials to exhibit complex, interconnected multi-level pore structures, alongside negatively charged components to activate key coagulation factors and initiate the coagulation cascade pathway [11,12,13,14,15]. However, their hemostatic efficacy is still insufficient for controlling bleeding in dynamic and challenging environments, such as non-compressible hemorrhage, surgical procedures, and patients with coagulation disorders. To solve these problems, hemostatic agents, including peptides such as γ-polyglutamic acid and poly(L-lysine) or thrombin, are commonly used to rapidly promote platelet aggregation and activation, thereby facilitating the conversion of fibrinogen into insoluble fibrin, enhancing the hemostatic effect [16,17,18,19,20]. But, the high cost, potential toxicity, risk of viral transmission, and other limitations have hindered widespread use. Therefore, active ingredients with excellent hemostatic performance, good biocompatibility, and cost-effectiveness hold extremely high prospects for clinical application.

Galla chinensis, a traditional Chinese herbal medicine, exhibits a unique hemostatic function and antibacterial, antioxidant, and wound-healing capabilities [21,22,23]. The polyphenols in Galla chinensis extract, such as tannic acid, gallic acid, and chlorogenic acid, can rapidly undergo cross-linking reactions with blood proteins and wound tissues, and subsequently activate the endogenous coagulation pathways and effective hemostasis. Meanwhile, in a moist environment, Galla chinensis can achieve significant antibacterial effects by interfering with bacterial metabolism and reducing their activity [24,25,26,27]. But the poor solubility of the water extract of Galla chinensis in the blood leads to the presence of large quantities that prevent effective hemostasis and create additional burdens on the liver and kidneys, thereby limiting its use in hemostasis applications. However, at present, there is still a lack of effective strategies to ensure the hemostatic and antibacterial efficacy of Galla chinensis extracts while minimizing the negative effects of drug overdose or excessively high local concentrations.

In this paper, a novel hemostatic and antibacterial granule loaded with Galla chinensis extract (GC) was developed. Firstly, mesoporous silica (MS) and calcium alginate were used to fabricate composite hemostatic granules (CMS) featuring an in situ porous structure using liquid-phase molding technology. Subsequently, uniformly, Galla chinensis extract was effectively loaded by physical adsorption onto the large-surface-area porous structure (CMS-GC) using the impregnation method. The surface of the CMS-GC granules is abundant in negatively charged alcohol hydroxyl groups, which can rapidly release Ca^2+^ and thereby activate the endogenous coagulation cascade reaction at the wound site. Meanwhile, the GC delivered from the CMS-GC granules combines with the plasma proteins to establish a 3D network structure, thereby facilitating the formation of a stable blood clot and accelerating hemostasis. In addition, the alginate maintains a moist environment, enabling GC to fully perform its functions of scavenging free radicals, enhancing antibacterial effects, and accelerating wound healing. Both in vitro and in vivo experimental results have demonstrated that CMS-GC granules exhibit distinctive rapid hemostatic and antibacterial properties. These properties enable efficient healing subsequent to tissue trauma bleeding and offer a novel therapeutic approach for clinical wound management.

## 2. Materials and Methods

### 2.1. Materials

Calcium carbonate (20011062) was obtained from Sinopharm Chemical Reagent Co., Ltd. (Shanghai, China). Silicon dioxide powder was provided by Shanghai Green Strong New Materials Co., Ltd. (Shanghai, China). Sodium alginate (S100127, Aladdin) was purchased from Hangzhou Gaosheng Biotechnology Co., Ltd. (Hangzhou, China). Chinese herbal Galla chinensis extract was acquired from Xi’an Wuyecao Biotechnology Co., Ltd. (Xi’an, China).

### 2.2. Preparation of the CMS-GC Hemostatic Granules

Based on our group’s previous work, the CMS granules were prepared using 1.0 g mesoporous silica (MS), 0.1 g calcium carbonate (CaCO_3_), and sodium alginate (SA) at four different levels, 0.025, 0.05, 0.075, and 0.1 g, to optimize slurry viscosity, which affects granule formation and water absorption. For each formulation, the reagents were transferred into a 50 mL centrifuge tube, followed by the addition of 20 mL ultrapure water. The mixture was homogenized with agate beads to obtain a uniform slurry. Each slurry was then added dropwise into hydrochloric acid (HCl, pH = 1) solution to achieve in situ pore formation in the liquid-phase system. The resulting granules were subjected to in-situ curing for 2 h to ensure complete formation of the calcium–alginate network and to allow the generated CO_2_ to gradually escape, producing the interconnected porous structure. After curing, the granules were repeatedly washed with ultrapure water until the wash solution reached neutrality and then freeze-dried to obtain the hemostatic granules denoted as CMS.

CMS granules (200 mg) were weighed and immersed separately in 1 mL of Galla Chinensis extract solutions at concentrations of 22.22, 35.29, 50.00, and 66.67 mg/mL, corresponding to theoretical drug loadings of 10%, 15%, 20%, and 25%, respectively. After immersing for 30 min, the granules were collected by filtration and freeze-dried. The resulting products were denoted as CMS-10%GC, CMS-15%GC, CMS-20%GC, and CMS-25%GC, respectively, based on their targeted theoretical drug loadings.

### 2.3. UHPLC-Q Exactive-MS Analysis of GC Components in GC

Analysis was performed on an Ultimate 3000 UHPLC-Q Exactive MS system (Thermo Fisher Scientific, Waltham, MA, USA). Separation used a Welchrom C18 column (100 × 2.1 mm, 1.8 μm) at 30 °C with a mobile phase of (A) 5 mM ammonium formate + 0.1% formic acid in water and (B) acetonitrile, at 0.3 mL/min. Gradient: 0–2 min, 100% A; 2–25 min, 100–5% A; 25–30 min, 100% A. The sample (5 mg Galla chinensis extract) was dissolved in 50% methanol, filtered (0.22 μm), and injected (5 μL). MS was operated in Full MS/dd-MS^2^ (Top 10) mode with HESI in the positive and negative modes, resolutions of 70,000 (MS) and 17,500 (MS^2^), and stepped HCD energies (15, 30, 45 eV). Compounds were tentatively identified by matching with the OTCML and GNPS spectral libraries.

### 2.4. Quantification of Total Polyphenol Content in GC

The total polyphenol content in GC was determined using the Folin–Ciocalteu colorimetric method. Gallic acid was used as the standard, and a series of standard solutions were prepared. An aliquot of 200 μL of Folin–Ciocalteu reagent was transferred and mixed with 40 μL of the GC test solution (or gallic acid standard solution) and 200 μL of 15% (*w*/*v*) Na_2_CO_3_ solution, and then vortexed and incubated for 30 min. The absorbance was recorded at 760 nm using a UV-Vis spectrophotometer (Shanghai INESA Analytical Instrument Co., Ltd. (Shanghai, China)).

### 2.5. Characterizations of CMS-GC Hemostatic Granules

The hemostatic granules were freeze-dried for 48 h, and then examined by a scanning electron microscope (SEM) and Fourier transform infrared spectroscopy (FTIR). Nitrogen adsorption–desorption (BET) was used to characterize their pore characteristics. Thermogravimetric analysis (TG) together with degradation assays was carried out to assess mass loss and degradation behavior.

Drug Loading Assay: The filtrate obtained after the immersion procedure described in Section 2.2 was collected. The concentration of un-adsorbed GC extract in the filtrate was determined by high-performance liquid chromatography (HPLC). The drug loading content (DLC) and encapsulation efficiency (EE) were calculated using the following equations:

(1) $$DLC\left(\%\right)=\frac{Total\;amount\;of\;GC-Free\;amount\;of\;GC}{Weight\;granules}\times 100\%$$

(2) $$EE\left(\%\right)=\frac{Total\;amount\;of\;GC-Free\;amount\;of\;GC}{Total\;amount\;of\;GC}\times 100\%$$

Drug Release Assay: Weigh 100 mg of hemostatic granules and place them into 4 mL of deionized water. Conduct the release experiment under constant shaking at 37 °C. At predetermined time intervals, withdraw 1 mL of the release medium, dilute it, and filter it through a 0.22 μm membrane filter for analysis. The concentration of GC in the release medium was determined by high-performance liquid chromatography (Waters E2695, Milford, MA, USA). The chromatographic conditions were as follows: mobile phase A was 0.1% formic acid in water and mobile phase B was acetonitrile, with a gradient elution (0–2 min, 90% A; 2–13 min, 90%→10% A; 13–15 min, 90% A); detection wavelength was 270 nm; flow rate was 1 mL/min; injection volume was 10 μL; column temperature was 30 °C. Linear regression of peak area versus concentration was performed to calculate the GC content in the samples.

### 2.6. Analysis of Fluid Absorption Ability

A total of 100 mg of hemostatic granule was weighed and transferred to a plastic centrifuge tube, with its exact mass recorded as W_0_. For water absorption testing, the granules were completely immersed in 1 mL of deionized water. At 5 s, 10 s, 30 s, 60 s, 120 s and 180 s, the deionized water was carefully removed to reweighing the material; its mass was recorded as W. For blood absorption testing, the granules were soaked in rat whole blood for 30 min until saturated, followed by reweighing the material; its mass was recorded as W. The water absorption capacity and blood absorption capacity of the hemostatic granules were calculated using the respective formulas below:

(3) $$Fluid\;absorption\;rate\;\left(\%\right)=\frac{W-W_{0}}{W_{0}}\times 100\%$$

### 2.7. Determination of Coagulation Time

To initiate coagulation, 30 μL of 0.025 mol/L CaCl_2_ solution was gently mixed with 270 μL of citrated whole blood using a pipette. Subsequently, 150 μL of the activated blood sample was transferred onto 10 mg of hemostatic granules placed in a centrifuge tube. Coagulation time was determined by a blinded observer using a visual tilt test, with the tube tilted gently every 1–2 s to monitor blood fluidity. Coagulation time was recorded when the blood lost fluidity and formed a solid clot.

### 2.8. In Vitro Evaluation of Coagulation-Promoting Ability via Blood Clotting Index (BCI)

Firstly, 100 μL of CaCl_2_-activated rabbit blood was spotted onto 100 mg of hemostatic granules. After 5 min, 20 mL ultrapure water dissolved uncoagulated blood in the system, and the supernatant’s 540 nm absorbance was measured via a microplate reader (OD_sample_). For the reference group, 100 μL of the same CaCl_2_-activated rabbit blood was mixed with 20 mL of ultrapure water (without hemostatic granules), and its absorbance was denoted as OD_reference_. The following formula was used to calculate the coagulation index of hemostatic granules:

(4) $$BCI\;\left(\%\right)=\frac{{OD}_{sample}}{{OD}_{reference}}\times 100\%$$

### 2.9. PT and aPTT of CMS-GC Hemostatic Granules

A semi-automatic blood coagulation analyzer was used to determine clinically relevant coagulation parameters, including prothrombin time (PT) and activated partial thromboplastin time (aPTT). Citrated whole blood was centrifuged at 3000 rpm for 10 min to obtain platelet-poor plasma (PPP). Subsequently, 200 μL of PPP was incubated with 10 mg of hemostatic granules at 37 °C for 10 min. Thereafter, the four coagulation parameters (PT and aPTT) were sequentially measured using the semi-automatic blood coagulation analyzer in accordance with the manufacturer’s standard operating procedures.

### 2.10. Evaluation of Blood Cell Adhesion

A total of 200 μL red blood cell (RBC) suspension was mixed with 100 mg hemostatic granules, followed by incubation at 37 °C for 1 h. Non-adherent RBCs were removed by washing with PBS, and the granules were then placed into 5 mL deionized (DI) water to induce RBC lysis. The absorbance of the lysate at 540 nm was documented as OD_sample_. For the reference group, 200 μL of the identical RBC suspension was directly lysed in 5 mL DI water (OD_reference_), and the RBC adhesion rate was then calculated according to the specified formula:

(5) $$RBCs\;adhension\;rate\left(\%\right)=\frac{{OD}_{sample}}{{OD}_{reference}}\times 100\%$$

Another portion of hemostatic granules (gauze as the control) was mixed with 100 μL fresh rat whole blood and incubated at 37 °C for 15 min. Samples were fixed and dehydrated after rinsing off unattached blood cells with PBS, and then photographed via SEM.

### 2.11. Calcium-Dependent Coagulation Assay

Platelet-poor plasma (PPP) was obtained by centrifuging citrated SD rat blood. The plasma was co-incubated with CMS and CMS-GC hemostatic granules at 37 °C, and clotting was observed by tilting the tube. A separate recalcification group was set up by adding 0.025 mol/L CaCl_2_ solution, mixed, and incubated at 37 °C for observation. A visible gel-like clot was taken as evidence of coagulation.

### 2.12. Evaluation of Protein Adsorption Performance of CMS-GC Hemostatic Granules

A total of 100 mg of hemostatic granules was weighed and pre-immersed in PBS buffer for 5 min. After removal from PBS, the granules were weighed again, and this mass was recorded as the initial mass. The granules were then transferred into 10 mL of 0.2 wt% bovine serum albumin (BSA) solution and incubated on a shaker for 30 min. After incubation, the absorbance at 280 nm of the initial BSA solution and the supernatant after adsorption was measured using a UV spectrophotometer (Shanghai INESA Analytical Instrument Co., Ltd. (Shanghai, China)). Meanwhile, an equal amount of granules was immersed in 10 mL of PBS, and the absorbance of the resulting supernatant at 280 nm was measured as a background control to eliminate interference from drug release. The protein adsorption capacity (mg/g) of the granules was calculated according to the following equation:

(6) $$Absorbed\;BSA\;(mg/g)=\frac{C_{0}-C_{a}}{W}V$$

C_0_ and C_a_ represent the concentrations of BSA in the solution before and after adsorption, respectively; W represents the mass of the granules (g); and V represents the volume of the BSA solution (mL).

### 2.13. Assessment of Antibacterial Properties

Hemostatic granules were immersed in sterile LB broth to prepare extracts. A total of 200 μL bacterial cultures of Gram-negative *Escherichia coli* (*E. coli*, BNCC269342) and Gram-positive *Staphylococcus aureus* (*S. aureus*, BNCC340652) were inoculated into LB medium, incubated at 37 °C for 6 h, and then diluted under sterile conditions to 1 × 10^6^ CFU/mL with liquid medium. The bacterial suspensions were then co-incubated with hemostatic granule extracts for 6 h. After co-culture, 100 μL bacterial suspensions were 10^4^-fold diluted, plated on solid LB agar, and incubated at 37 °C. Following incubation, colony formation was photographed and counted to calculate the bactericidal rate based on CFU enumeration. The absorbance at 600 nm was also measured as a complementary method for calculating the bactericidal rate. In addition, to meet international standards, the following ATCC reference strains were incorporated for benchmark testing: *E. coli* (ATCC 25922), *S. aureus* (ATCC 25923), and *methicillin-resistant Staphylococcus aureus* (*MRSA*, ATCC 43300). The antibacterial activity of the hemostatic granules against these strains was evaluated using the spread plate method. To determine the antibacterial effect of the hemostatic granule extracts, the relative ATP content of bacteria co-cultured with the granule extracts was assessed using an ATP assay kit (Beijing RuiDaHengHui Science & Technology Development Co., Ltd. (Beijing, China)).

The bacterial biofilm inhibitory effect of hemostatic granule extracts was assessed via crystal violet staining. A bacterial suspension of 10^7^ CFU/mL was co-cultured with the test material’s extract in a 24-well plate to induce biofilm formation. After washing non-adherent bacteria with PBS, samples were stained with 0.1% crystal violet for 30 min (photographed for morphology). Post-decolorization with 95% ethanol, the eluted crystal violet’s 570 nm OD value was measured via a microplate reader to reflect the biofilm’s relative biomass.

For bacterial morphological analysis, *E. coli* (1 × 10^7^ CFU/mL) and *S. aureus* (1 × 10^7^ CFU/mL) were co-cultured with the hemostatic granule extracts for 4 h. The bacteria were fixed and dehydrated before imaging. After ultrasonic dispersion, 20 μL of bacterial suspension was pipetted onto silicon wafers and examined using SEM.

### 2.14. In Vivo Hemostatic Efficacy Evaluation

The hemostatic efficacy of CMS-15%GC was evaluated in rat models of liver and femoral artery hemorrhage, using CMS and gauze as controls. Rats were anesthetized via intraperitoneal injection of sodium pentobarbital, and the surgical sites were shaved.

For the liver hemorrhage model, a laparotomy was performed to expose the liver, and pre-weighed filter paper was placed underneath. A 1 cm transverse incision was made on the liver surface with a scalpel. After allowing free bleeding for a few seconds to establish a consistent baseline, 100 mg of each hemostatic material (CMS-15%GC, CMS, or gauze) was immediately applied to the wound. Photographs were taken at 10, 30, 60, 120, and 180 s post-application, and the bleeding time and total blood loss were recorded.

For the femoral artery hemorrhage model, the femoral artery was carefully isolated and completely transected with surgical scissors. After free bleeding for 3 s to ensure a consistent baseline, 100 mg of each material was immediately applied to the injury site. Hemostasis time and total blood loss were recorded.

### 2.15. Determination of Antioxidant Properties

The antioxidant capacity of the hemostatic granules was assessed using DPPH and ABTS free radical scavenging assays. The working solutions of DPPH and ABTS were first prepared. GC and hemostatic granules (10 mg each) were separately added to the DPPH/ABTS solutions and incubated in the dark for 30 min. Then, supernatants were collected to measure absorbance at 517 nm (DPPH) and 734 nm (ABTS) via ultraviolet spectrophotometer. Free radical scavenging capacity was calculated using the specified formula:

(7) $$Free\;radical\;scavenging\;activity\;\left(\%\right)=\frac{A_{0}-A_{1}}{A_{0}}\times 100\%$$

A_0_ represents solution without adding materials, and A_1_ represents solution with adding materials.

### 2.16. Determination of Biocompatibility

L929 cells were cultured in DMEM medium containing 10% serum. For granule extract preparation, 100 mg of lyophilized hemostatic granules were precisely weighed, sterilized under UV light overnight, and then immersed in 1 mL DMEM and incubated for 24 h to obtain extract. Biocompatibility was assessed via CCK8 assay and Live/Dead staining: in the CCK8 assay, L929 cells were seeded in 96-well plates at a density of 1 × 10^5^ cells per well, incubated at 37 °C for 24 h to adhere, and then cultured with the granule extract for another 24 h; post-incubation, the extract was removed, cells were washed with PBS, CCK8 solution was added, and absorbance at 450 nm was measured for viability calculation. For Live/Dead staining, cells were plated in 24-well plates at 3 × 10^5^ cells per well, allowed to adhere for 24 h, cultured with 0.1 g/mL extract for 24 h, stained with Live/Dead kit, and observed under laser confocal microscopy to evaluate morphology and survival.

To evaluate the in vivo biocompatibility of CMS-15%GC, a subcutaneous implantation model was developed in SD rats. After anesthesia, the dorsal area was shaved and sterilized. A longitudinal incision was made on each side of the spine, and subcutaneous pockets were created using blunt dissection. CMS-15%GC hemostatic granules were implanted into the pockets, and the incisions were closed with sutures. The rats were euthanized on days 7 and 14 post-implantation, and tissue samples were collected from the implantation sites and adjacent normal skin for hematoxylin and eosin (HE) staining.

### 2.17. Analysis of the Hemolytic Properties

A total of 10 mg test material was added to 800 μL PBS and mixed gently with 200 μL 5% (*v*/*v*) RBC suspension. Controls: PBS (negative, no hemolysis) and ultrapure water (positive, complete hemolysis). All mixtures were incubated for 1 h, and then, 100 μL of each was transferred to a 96-well plate; 540 nm absorbance was detected to calculate hemolysis percentage using the formula:

(8) $$\mathrm{Hemolysis}\;\mathrm{ratio}\;(\%)\;=\;\frac{{OD}_{sample}-{OD}_{negative\;control}}{{OD}_{positive\;control}-{OD}_{negative\;control}}\times 100\%$$

### 2.18. Evaluation of Platelet Toxicity

A total of 500 μL platelet-rich plasma (PRP) was thoroughly mixed with 500 μL hemostatic granule extract, incubated at 37 °C for 2 h, centrifuged at 400× *g* for 5 min, and the supernatant’s lactate dehydrogenase (LDH) activity was measured via assay kit (OD_sample_). Controls: PRP + 1 mL 1% Triton X-100 (positive, complete platelet lysis, OD_reference_) and pure PBS (negative). Platelet toxicity was calculated using the specified formula:

(9) $$LDH\;activity\left(\%\right)=\frac{{OD}_{sample}}{{OD}_{reference}}\times 100\%$$

### 2.19. In Vivo Wound Healing Test

All procedures were approved by Shanghai Chengxi Biotechnology Co., Ltd. (approved number: CX052512110) and conducted in accordance with the “3Rs” principle and relevant provisions of the national Guiding Opinions on the Humane Treatment of Laboratory Animals.

A full-thickness *S. aureus*-infected skin defect model was established in male SD rats (200–250 g): rats were anesthetized by intraperitoneal injection of sodium pentobarbital, dorsal hair was removed, and 10 mm diameter, 2 mm deep skin wounds were created with sterile surgical scissors; 50 μL of *S. aureus* suspension (1 × 10^6^ CFU/mL) was inoculated onto the wounds and incubated for 24 h to confirm infection. Approximately 50 mg of hemostatic granules (CMS or CMS-15%GC) were applied to fully cover the wounds (untreated group without granules), followed by gauze and medical bandage dressing. The granules were replaced daily, and the wound photographs were captured on days 0, 3, 7, 10, and 14, with areas quantitatively analyzed using the ImageJ software (version 1.54g). To assess wound healing-related histological changes and immune responses, skin tissues from the defect sites were collected after rats were euthanized, sectioned, and subjected to hematoxylin–eosin (HE) staining and Masson staining for evaluating healing quality and collagen deposition microstructure; major organs (heart, liver, spleen, lung, kidney) were also harvested at the end of the experiment to observe potential pathological changes.

For exploring the immune regulation mechanism within regenerated cutaneous tissue, immunofluorescence staining was conducted: on day 3, excised regenerated skin tissues were stained for the inflammatory factor TNF-α and pan-macrophage marker CD68; on day 7, staining was performed for neovascularization markers CD31, and α-smooth muscle actin (α-SMA).

### 2.20. Statistical Analysis

Experimental data were analyzed using GraphPad Prism 10. For in vitro assays, each experiment was independently repeated at least three times (technical replicates). For in vivo experiments, each group consisted of *n* = 4 rats (biological replicates). Data are presented as mean ± standard deviation (SD). Inter-group differences were analyzed by one-way ANOVA followed by Tukey’s multiple comparisons test, with error bars representing SD. Statistical significance was set at * *p* < 0.05, ** *p* < 0.01, *** *p* < 0.001, **** *p* < 0.0001.

## 3. Results and Discussion

### 3.1. Characterization of the CMS-GC Hemostatic Granules

**Scheme 1:**
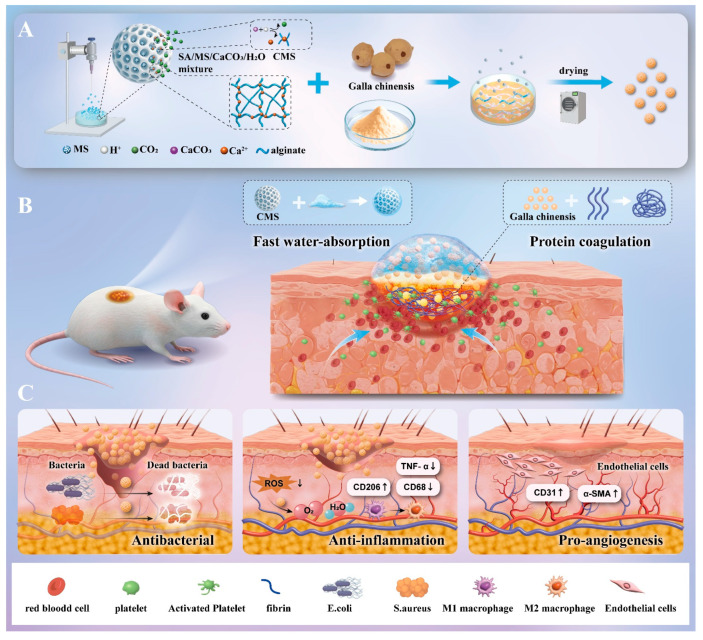
Preparation of hemostatic granules and their biological application in hemostasis. (**A**) Preparation process and formation mechanism of CMS-GC hemostatic granules. (**B**) The application of CMS-GC hemostatic granules. (**C**) Explanation of the potential mechanism of multifunctional CMS-GC hemostatic granules in healing infected wounds.

CMS-GC hemostatic granules were prepared by mixing calcium carbonate, MS, and SA at a defined mass ratio to obtain a homogeneous slurry. The slurry was subsequently added dropwise into a hydrochloric acid solution. During this process, CaCO_3_ reacts with HCl to release Ca^2+^ ions, which progressively crosslink alginate chains through ionic interactions according to the classical “egg-box” model, leading to the formation of a stable calcium–alginate gel network [28]. Meanwhile, CO_2_ bubbles generated during the reaction act as a porogen, thereby generating an interconnected porous structure in situ and facilitating granule formation and structural stabilization, ultimately yielding CMS hemostatic granules. After freeze-drying, the granules were subjected to swelling adsorption to load the extract of Galla chinensis (GC) onto the CMS, which was followed by a second freeze-drying step (Scheme 1A). The CMS granule formulation was established based on previous formulations developed by our research group. The quantities of MS (1 g) and CaCO_3_ (0.1 g) were kept constant, whereas the SA content was systematically varied to evaluate its effect on granule formation. Water absorption after 30 s was measured as a preliminary indicator of rapid uptake, reflecting potential hemostatic efficacy (Figure S1). At an SA content of 0.025 g, stable granules were not formed, resulting in relatively low water absorption. At higher SA contents (0.075 and 0.1 g), enhanced crosslinking during solidification produced harder granules, thereby decreasing water uptake. Based on these observations, an SA content of 0.05 g was selected, as it provided an optimal balance of granule formation, morphology, stability, and rapid water absorption.

To elucidate the chemical composition of Galla chinensis (GC) extract and understand the potential bioactive components contributing to its hemostatic and antibacterial effects, UHPLC-Q Exactive-MS analysis was performed, leading to the tentative identification of several representative compounds, including abscisic acid, pyrogallol, quinic acid, 4-hydroxybenzoic acid, osthole, gallic acid, and ellagic acid, by matching with the OTCML and GNPS databases (Figure S2). Detailed information, including the retention times and molecular formulas of the identified compounds, is summarized in Table S1. Previous studies have reported that gallic acid exhibits antioxidant, antibacterial, and anti-inflammatory activities, which may contribute to infection control and modulation of the wound microenvironment [29]. Ellagic acid has also been associated with hemostatic activity and may participate in promoting clot formation [30]. In addition, other polyphenolic compounds identified in GC, such as pyrogallol and 4-hydroxybenzoic acid, may provide auxiliary bioactivities, including antibacterial and redox-regulating effects, thereby collectively contributing to the multifunctional performance of CMS-GC granules. The content of gallic acid in GC, calculated based on the standard curve of gallic acid, was 17.24 ± 0.95 μg/g (Figure S3). The total polyphenol content of the GC was determined to be 8.922 ± 0.566% using the Folin–Ciocalteu method (Table S2). In addition, the GC extract used in this study was obtained from a commercial supplier and accompanied by a certificate of analysis. Nevertheless, natural plant extracts may exhibit compositional variability depending on the botanical source, harvesting conditions, and extraction process, which may affect the reproducibility of biomaterial performance. Therefore, future studies will further strengthen GC extract standardization and quality-control strategies to improve batch-to-batch consistency and translational reliability.

**Figure 1:**
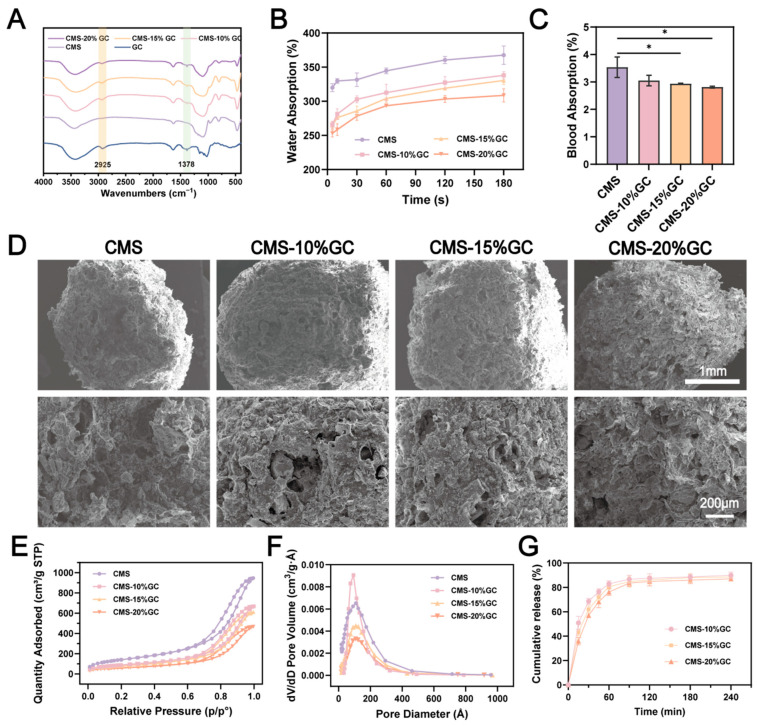
Physicochemical characterization of CMS and CMS-GC hemostatic granules. (**A**) FTIR spectra of CMS and CMS-GC granules. (**B**) Water absorption capacity measured gravimetrically after immersion in deionized water. (**C**) Blood absorption capacity was evaluated using sodium citrate-anticoagulated fresh whole blood to assess fluid uptake relevant to hemostatic performance. (**D**) SEM images showing the surface morphology and porous microstructure of the granules. (**E**) Nitrogen adsorption–desorption isotherms for analysis of specific surface area and pore structure. (**F**) Pore size distribution calculated using the BJH method. (**G**) Cumulative release profile of loaded active components measured in vitro (n = 3 independent experiments). (* *p* < 0.05).

The FTIR spectra of CMS, MS, and SA are shown in Figure S2. In the CMS spectrum, the broad absorption band at 3461 cm^−1^ corresponds to -OH stretching vibrations, indicating the presence of hydroxyl groups from SA and adsorbed water. The peak at 1021 cm^−1^ is assigned to C-O-C stretching, characteristic of the polysaccharide backbone of SA. Peaks at 1641 cm^−1^ and 1414 cm^−1^ correspond to the asymmetric and symmetric stretching of carboxylate anions in SA, confirming the preservation of carboxyl functionality after integration with MS. The broad Si-O-Si absorption at 1079 cm^−1^ reflects the presence of MS, partially overlapping with the C-O-C and -COO^−^ peaks from SA. Overall, the CMS spectrum demonstrates the coexistence of the MS framework and SA-derived functional groups, indicating successful formation of the CMS composite with preserved SA chemical structure. As shown in the infrared spectrum, compared with CMS, all the CMS-GC samples exhibited characteristic absorption peaks at 1378 cm^−1^ and 2925 cm^−1^, thereby confirming their successful preparation (Figure 1A and Figure S5). The peak at 2925 cm^−1^ corresponds to C-H stretching in GC, while the characteristic peak at 1378 cm^−1^ arises from coupling between O-H bending, C-H bending, and ring stretching vibrations [31]. Furthermore, slight shifts and broadening of the -OH and C-H peaks in CMS-GC compared with the individual CMS and GC spectra suggest that GC is physically adsorbed onto the CMS matrix, indicating weak non-covalent interactions between GC molecules and the silica–alginate framework.

In vitro water absorption tests demonstrated that, within 10 s, the absorption rate of all hemostatic granules exceeded 250% (Figure 1B), indicating their ability to rapidly absorb water in the initial stage and thereby concentrate blood components. The elevated concentrations of clotting factors and platelets rapidly activated the coagulation cascade, resulting in fibrin deposition and clot formation, thereby achieving efficient hemostasis. However, with the increase in drug loading, both the water absorption rate and the blood absorption rate decreased (Figure 1C and Figure S6), possibly because of partial blockage of the pore structure by GC. SEM images revealed large pit-like structures on the surface of the hemostatic granules, enabling rapid water absorption during the initial stage (Figure 1D). To quantify the granule size, fifty granules from each sample were randomly measured, and their size distributions are presented in Figure S7. The mean diameters were 2.93 ± 0.46 mm for CMS, 2.88 ± 0.41 mm for CMS-10%GC, 2.96 ± 0.45 mm for CMS-15%GC, and 2.91 ± 0.40 mm for CMS-20%GC. All samples exhibited unimodal distributions, with the majority of granules ranging from 2.4 to 3.2 mm, indicating relatively uniform particle sizes. These results are consistent with previously reported values for similar alginate–silica granules, confirming that the granulation method yields reproducible, well-defined hemostatic particles suitable for practical applications [32]. Nitrogen adsorption–desorption analysis (Figure 1E and Table S3) revealed that the granules possessed an interconnected porous structure, with the specific surface area decreasing from 509.13 m^2^/g for CMS to 216.57 m^2^/g for CMS-20%GC, likely due to the partial occupation of pores by GC. The total pore volume showed a corresponding decrease. The pore size distribution of the CMS-GC granules was analyzed using the BJH method based on the desorption branch (Figure 1F), which provides a reasonable assessment of mesoporous features. Although this method does not fully capture microporous or macroporous structures, it is sufficient to describe the mesopore range relevant to the hemostatic performance of these granules. These mesoporous structures provide accessible surface area and pore volume, which may promote the rapid infiltration of water and plasma proteins, facilitate the concentration of platelets and coagulation factors, and support the formation of an initial stable clot.

Additionally, the drug loading capacities of CMS-10%GC, CMS-15%GC, and CMS-20%GC were 9.34% ± 0.11%, 13.40% ± 0.26%, and 16.52% ± 0.33%, respectively, while the encapsulation efficiencies were 92.73% ± 1.21%, 87.67% ± 1.94%, and 79.18% ± 1.95%, respectively (Figure S8). To further assess the GC loading in the CMS granules, thermogravimetric analysis (TGA) was conducted on hemostatic granules (Figure S9). All samples exhibited two distinct weight loss regions. The first region, occurring in the range of approximately 50–150 °C, corresponded to the removal of physically adsorbed and bound water. The second weight loss, observed in the range of 200–600 °C, resulted from the thermal decomposition of the calcium alginate matrix in CMS. In the CMS-GC samples, this second weight loss also encompassed the decomposition of the loaded GC, leading to a greater mass loss relative to CMS. Based on the difference in mass loss between CMS and CMS-GC within this temperature range, the GC loading was estimated to be approximately 8.79%, 12.08%, and 15.29% for CMS-10%GC, CMS-15%GC, and CMS-20%GC, respectively, consistent with the values previously determined via HPLC analysis. The results indicate that the actual GC loading increased progressively with the theoretical drug amount, demonstrating that the CMS granules possess a substantial capacity to accommodate GC. Conversely, the EE exhibited a decreasing trend, likely due to the progressive saturation of binding sites on the surface and within the pores of the granules. At higher theoretical loadings, a fraction of GC molecules may not be fully adsorbed by CMS, resulting in reduced EE. These observations suggest that, although CMS granules can effectively load GC, there exists an upper limit to the amount that can be efficiently encapsulated, consistent with the TGA-derived estimations of drug content. Drug release experiments revealed that nearly 40% of the GC was released within 15 min, and this burst release satisfied the requirement for rapid hemostasis in the initial stage. After 2 h, the cumulative release of GC exceeded 85% (Figure 1G). The prolonged release over 2 h is likely due to interactions between GC and the CMS matrix, which contribute to sustained release. This gradual release ensures effective hemostasis and antibacterial effects, while preventing rapid GC release and reducing the risk of toxicity from systemic circulation. In this study, deionized water was used as the release medium to avoid interference from salts or buffer components during UHPLC quantification of GC. Water-based media have also been commonly used in preliminary polyphenol release studies, supporting the use of deionized water for initial release evaluation [33,34]. Although deionized water facilitates accurate quantification, it does not fully mimic physiological conditions and may therefore result in a slightly faster release rate than that observed in vivo. Future studies will employ PBS or plasma as release media to obtain release profiles that more closely reflect the in vivo environment.

The above results demonstrate the successful preparation of CMS-GC hemostatic granules, which exhibit rapid water absorption and blood component concentration, activate the coagulation system to achieve highly effective hemostasis, and display a GC release profile that meets the requirements for initial rapid hemostasis followed by sustained efficacy. However, the DLC of the granules affects their water absorption capacity, blood uptake performance, pore structure, and specific surface area, thus necessitating optimization by balancing drug loading with hemostatic efficacy.

### 3.2. Biocompatibility and Hemostatic Performance of Hemostatic Granules In Vitro

**Figure 2:**
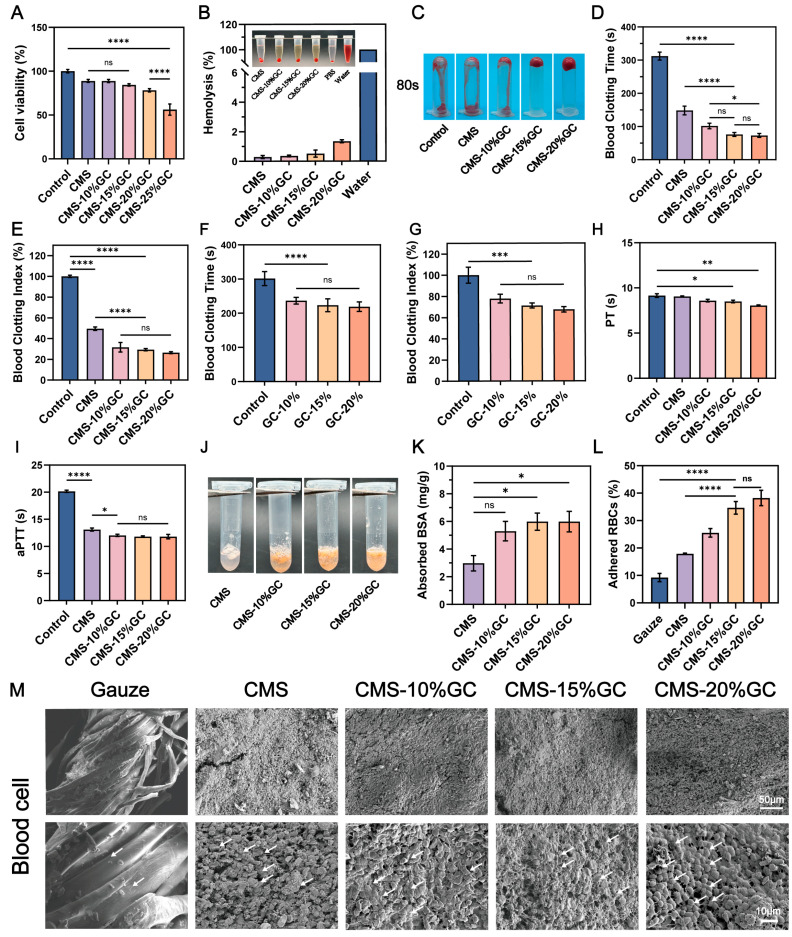
In vitro biocompatibility and hemostatic performance of CMS-GC hemostatic granules. (**A**) Viability of L929 cells treated with the CMS-GC hemostatic granule extract. (**B**) Hemolytic assay of CMS-GC hemostatic granules. (**C**) Representative images of hemostasis mediated by CMS-GC hemostatic granules in vitro. (**D**) In vitro clotting time measured after treatment with CMS-GC hemostatic granules. (**E**) Blood clotting index (BCI, %) of CMS-GC hemostatic granules, indicating clot formation efficiency. (**F**) In vitro clotting time measured after treatment with the drug GC. (**G**) Clotting index of GC drug as a comparison for hemostatic performance. (**H**) Prothrombin time (PT) of CMS-GC hemostatic granules measured in vitro. (**I**) Activated partial thromboplastin time (aPTT) of CMS-GC hemostatic granules measured in vitro. (**J**) Representative image showing the interaction between CMS-GC hemostatic granules and plasma. (**K**) Quantitative analysis of the adsorption performance of CSM-GC hemostatic granules on BSA. (**L**) Percentage of red blood cells (RBCs) adhering to gauze and hemostatic granules. (**M**) SEM images showing blood cell adhesion on the surfaces of different hemostatic granules (n = 3 independent experiments). (* *p* < 0.05, ** *p* < 0.01, *** *p* < 0.001 and **** *p* < 0.0001.ns, not significant.)

The biocompatibility of hemostatic granules is a fundamental prerequisite for their potential clinical application. Therefore, we systematically evaluated the cytocompatibility of CMS-GC granules with varying loading contents at a concentration of 100 mg/mL. CCK8 assay results demonstrate that the viability of L929 cells cultured with extracts of CMS-10%GC, CMS-15%GC, and CMS-20%GC granules remained above 75%, indicating that cytocompatibility at this concentration was acceptable (Figure 2A). In contrast, CMS-25%GC exhibited cytotoxic effects. Consistently, live/dead staining further confirmed these findings, as the CMS-10%GC, CMS-15%GC, and CMS-20%GC groups exhibited predominantly green fluorescence, with negligible numbers of dead cells (Figure S10). Overall, above data confirmed that the CMS-10%GC, CMS-15%GC, and CMS-20%GC granules exhibited favorable cytocompatibility, whereas CMS-25%GC exceeded the cytotoxicity threshold and was unsuitable for further investigation.

To further evaluate the in vivo biosafety of the hemostatic granules, CMS-15%GC was subcutaneously implanted in SD rats, and tissue samples were harvested at 7 and 14 days post-implantation for histological evaluation (Figure S11). On day 7, mild inflammation and fibroblast proliferation were observed around the implantation site, consistent with a typical early foreign body response, while the epidermal and dermal architectures remained intact, with no evidence of necrosis or severe inflammation. By day 14, the tissue response had further subsided, and a thin, continuous fibrous capsule had formed around the material, with no evident inflammatory cell infiltration or necrosis in the surrounding tissue. These findings indicate that the hemostatic granules exhibit favorable in vivo biocompatibility. Nevertheless, these observations are limited to a 14-day implantation period, providing only preliminary insight into material–tissue interactions. The H&E staining results may suggest partial material degradation during this period, but they do not allow a comprehensive assessment of long-term degradation, potential silica retention or accumulation, or the risk of chronic inflammatory responses. Future studies are warranted to systematically investigate these aspects through extended implantation periods, quantitative evaluation of residual material, assessment of silica biodistribution and clearance, and histological monitoring of chronic inflammatory markers, thereby enabling a more comprehensive evaluation of the long-term biosafety and translational potential of CMS-GC granules.

Hemostatic materials must exhibit good blood compatibility to minimize the risk of hemolysis. Hemostatic granules were cocultured with red blood cell suspensions (10 mg/mL) for 1 h to assess the risk of hemolysis. Ultrapure water was used as the positive control, and phosphate-buffered saline (PBS) was used as the negative control. The hemolysis rates of all experimental groups did not exceed 5% (Figure 2B). Further coculture of hemostatic granules with platelets was performed to assess their platelet compatibility. LDH released from platelets incubated with Triton X-100 was used as a positive control. The results show that LDH release after coculture with hemostatic granules was comparable to the minimal release observed in the negative control group (PBS) (Figure S12). These results demonstrat that the hemostatic granules possessed favorable blood compatibility.

Coagulation time is one of the most direct and widely used indicators for evaluating hemostatic efficiency. The hemostatic performance of the different granules was assessed via in vitro coagulation assays. Compared with the blank control group (312 ± 10 s), the CMS group decreased to about 148 s. With increasing GC loading, the hemostasis time of CMS-15%GC and CMS-20%GC decreased to less than 80 s (Figure 2C,D). Procoagulant function was quantified through measurement of the blood coagulation index (BCI). A clearer supernatant indicates a stronger procoagulant effect of the granules [35]. After the addition of ultrapure water, the supernatants from the CMS-15%GC and CMS-20%GC groups exhibited increased clarity, indicating the formation of stable blood clots (Figure S13). The quantitative assessment further verified that the BCI value of the CMS-15%GC group after drug loading was approximately 20% lower than that of the CMS group (Figure 2E). In parallel, equivalent doses of free GC were tested under the same conditions in the BCT assay (Figure 2F), but no significant reduction in coagulation time was observed. In addition, the pure GC polyphenols exhibited limited procoagulant activity (Figure 2G), potentially due to their low hydrophilicity—a property that hinders their adequate dispersion in blood and thus reduces their procoagulant capacity. In conclusion, pure GC exhibited limited intrinsic hemostatic activity and was insufficient to achieve effective hemostasis when used alone. Although increasing GC loading reduced the porosity, specific surface area, and water absorption capacity of the composite granules, the overall hemostatic efficacy was paradoxically enhanced. The enhanced effect results from the synergistic combination of the porous CMS framework and the bioactive properties of GC. Specifically, the CMS architecture facilitated rapid blood infiltration and local enrichment of coagulation components, thereby creating a microenvironment that allowed GC to interact more effectively with concentrated blood factors. This synergistic mechanism effectively compensated for the reduced physical properties, ultimately resulting in enhanced hemostatic performance.

The prothrombin time (PT) and activated partial thromboplastin time (aPTT) assays were performed to further investigate the contact-induced activation of the plasma coagulation cascade by the hemostatic granules. Compared with the negative control, the aPTTs were significantly shortened for all the groups, whereas the PTs showed no statistically significant differences. However, the CMS-GC group did not show a further reduction in aPTT when compared with the CMS group alone (Figure 2H,I). These results indicate that the intrinsic coagulation pathway is effectively activated, primarily due to the polar silanol groups on the porous silica surface, as reported in previous studies [36,37].

Calcium-dependent coagulation assays demonstrated that both CMS and CMS-GC require Ca^2+^ ions for their procoagulant activity (Figure S14). Upon recalcification, CMS-GC granules formed a flocculent layer when mixed with plasma (Figure 2J), suggesting that GC may act as a protein flocculating agent, promoting protein cross-linking and the formation of a three-dimensional clot network [26,27]. Consistent with this observation, quantitative BSA adsorption analysis further showed that GC loading significantly enhanced protein adsorption compared with CMS (Figure 2K). These results indicate that CMS-GC promotes hemostasis through enhanced protein adsorption and Ca^2+^-dependent activation of the coagulation cascade.

Effective hemostasis and thrombus formation rely heavily on the aggregation and adhesion of red blood cells (RBCs), which exert their hemostatic effects through inherent biomechanical properties and interactions with platelets [38]. Therefore, we conducted an RBC adhesion assay to quantify the proportion of adherent RBCs and found that the adhesion rate increased with increasing GC loading levels (Figure 2L). The adhesion rates for CMS-15%GC and CMS-20%GC were 35.6 ± 3.32% and 38.24 ± 4.38%, respectively, exceeding those of both the gauze group and the nondrug-loaded group. This effect may be attributed to the release of GC from the granules, which interacts with blood proteins and peptides via its phenolic hydroxyl groups, thereby stimulating coagulation and promoting RBCs adhesion [39,40]. Further SEM characterization of blood cell adhesion on the material surface (Figure 2M) revealed sparse and scattered blood cells on the gauze surface, whereas the other groups exhibited numerous, irregularly, and tightly adhered blood cells, together with a cross-linked fibrin network. This observation can be attributed to the porous structure of the hemostatic granules, which enables rapid blood concentration and blood cell aggregation. Concurrently, GC polyphenols can form cross-linked networks with fibrin in the blood [26].

The superior hemostatic efficacy of CMS-GC can be attributed to the following synergistic mechanisms: (1) The porous structure of CMS granules with large surface pits enables rapid water absorption and blood concentration upon contact with the bleeding site, physically promoting hemostasis consistent with the enhanced clot formation observed in the BCI assay and the dense clot morphology; (2) The silanol groups on the MS surface activate the intrinsic coagulation pathway leading to significant aPTT shortening [36,37,41,42]; (3) GC enhances plasma protein adsorption on the material surface. Upon direct contact with plasma, CMS-GC forms a visible flocculent layer more rapidly than CMS, and the insoluble protein complexes generated further physically block the bleeding site and stabilize the clot structure. This is consistent with the accelerated clot formation and stable clot morphology observed in the clotting time assays [25].

### 3.3. Evaluation of Hemostatic Effect of CMS-GC Hemostatic Granules In Vivo

Liver hemorrhage and femoral artery puncture models were established to comprehensively evaluate the hemostatic performance of the granules under physiological conditions. Based on the in vitro hemostatic assays, the CMS-15%GC group was selected for subsequent in vivo evaluation, with medical gauze and CMS serving as control groups. Preliminary tests showed that UV irradiation sterilization, conducted prior to the in vivo experiments, did not adversely affect the hemostatic performance of CMS and CMS-15%GC, preliminarily confirming their compatibility with this sterilization method. Future studies should systematically evaluate their performance under other sterilization methods (e.g., ethylene oxide, gamma irradiation) and long-term stability under different storage environments.

**Figure 3:**
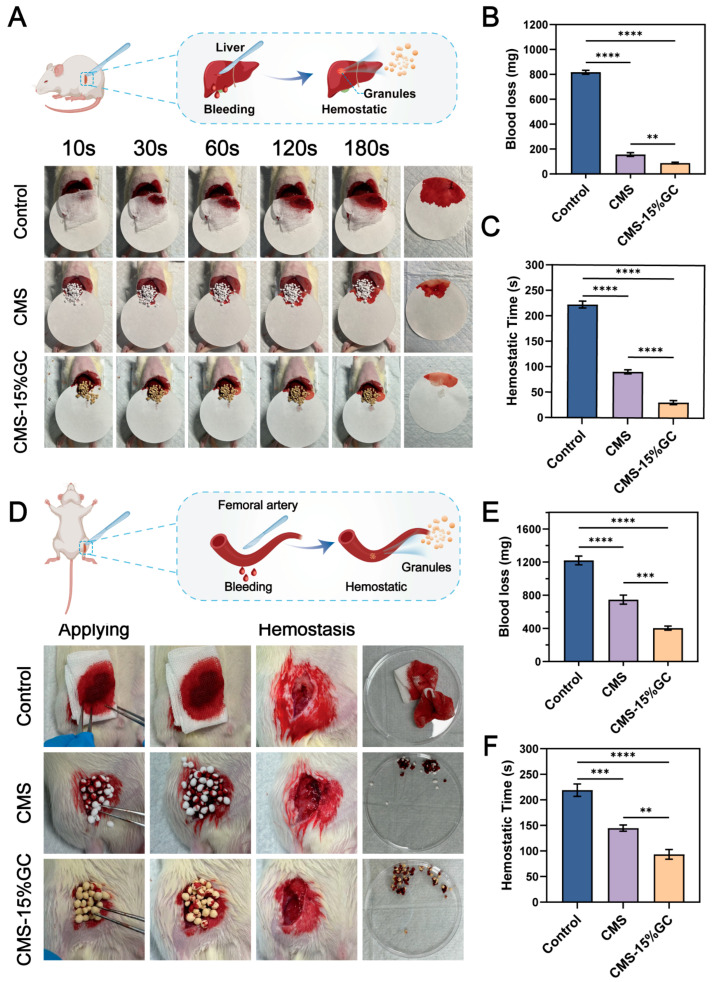
In vivo evaluation of the hemostatic efficacy of CMS-GC granules in rat bleeding models. (**A**) Schematic illustration of the rat liver hemorrhage model, showing the experimental setup and procedures for model establishment. (**B**) Quantitative analysis of total blood loss in the rat liver hemorrhage model following treatment with gauze (control), CMS, and CMS-15%GC granules. (**C**) Quantitative analysis of time to hemostasis in the rat liver hemorrhage model (n = 4 per group). (**D**) Schematic illustration of the rat femoral artery puncture model, showing the experimental design and procedures for model establishment. (**E**) Quantitative analysis of total blood loss in the femoral artery puncture model following treatment with different hemostatic materials. (**F**) Quantitative analysis of time to hemostasis in the femoral artery puncture model (n = 4 rats per group). (** *p* < 0.01, *** *p* < 0.001 and **** *p* < 0.0001.)

In the rat liver hemorrhage model, CMS-15%GC exhibited significantly reduced blood loss (88 ± 6 mg) and shorter hemostasis time (31 ± 3 s) compared with both the gauze group (817 ± 12 mg, 222 ± 6 s) and the CMS group (156 ± 13 mg, 90 ± 3 s) (Figure 3A–C). In the rat femoral artery puncture model, medical gauze failed to control bleeding (219 ± 10 s, 1220 ± 4 mg). Pure CMS demonstrated improved yet insufficient hemostatic performance (144 ± 5 s, 750 ± 5 mg). The hemostatic time of CMS-15%GC was only 93 ± 8 s, and the blood loss was reduced to 403 ± 20 mg (Figure 3D–F). In addition, we compared the hemostatic performance of CMS-15%GC with that of the commercial hemostatic agent Celox, as reported in the literature [43]. The comparison indicated that CMS-15%GC achieved superior outcomes, characterized by shorter hemostasis times and lower blood loss in both liver hemorrhage and femoral artery puncture models (Table S4).

In summary, these results demonstrate that the synergistic interaction between GC and CMS plays a critical role in enhancing hemostatic efficacy in both liver hemorrhage and femoral artery puncture models. These two models demonstrate the potential of CMS-15%GC to control life-threatening bleeding and improve clinical outcomes. It should be noted that the animal group sizes in the present study were relatively limited, which may reduce the statistical power and limit the biological generalizability of the in vivo findings. Therefore, although the current results show generally consistent hemostatic trends, further validation using larger animal cohorts is required to confirm the robustness of the material’s hemostatic performance. Furthermore, the two models employed in this study still cannot fully recapitulate complex clinical bleeding scenarios, such as high-pressure arterial bleeding, irregular wound surfaces with multiple bleeding points, or hemorrhage exacerbated by pre-existing coagulopathy such as hemophilia or anticoagulant use. Therefore, future studies should further validate the hemostatic efficacy of CMS-15%GC in larger cohorts and in more clinically relevant models, thereby providing a stronger preclinical foundation for its clinical translation.

### 3.4. Antioxidant and Antibacterial Properties of the Hemostatic Granules

**Figure 4:**
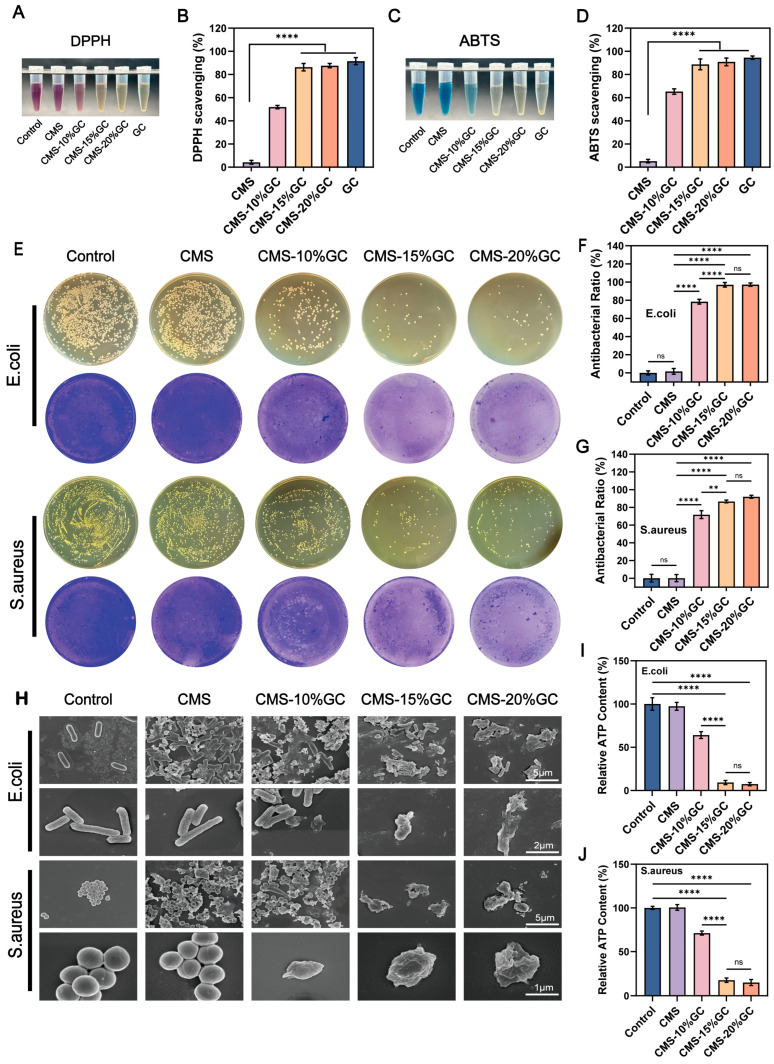
Evaluation of the antioxidant and antibacterial activities of CMS-GC hemostatic granules. (**A**) Representative images of the 2,2-diphenyl-1-picrylhydrazyl (DPPH) radical scavenging assay. (**B**) Quantitative analysis of DPPH radical scavenging activity based on absorbance at 517 nm. (**C**) Representative images of the 2,2′-azino-bis (3-ethylbenzothiazoline-6-sulfonic acid) (ABTS) radical scavenging assay. (**D**) Quantitative analysis of ABTS radical scavenging activity determined from absorbance at 734 nm. (**E**) Representative agar plate cultures and crystal violet staining images of *Staphylococcus aureus* (*S. aureus*) and *Escherichia coli* (*E. coli*) following treatment. (**F**) Quantitative analysis of antibacterial activity against *E. coli* based on colony-forming unit (CFU) counting. (**G**) Quantitative analysis of antibacterial activity against *S. aureus* based on CFU counting. (**H**) SEM images of *S. aureus* and *E. coli* after treatment to assess bacterial morphological alterations. (**I**) Intracellular adenosine triphosphate (ATP) levels in *E. coli* to evaluate bacterial metabolic activity. (**J**) Intracellular ATP levels in *S. aureus* to evaluate bacterial metabolic activity (n = 3 independent experiments). (** *p* < 0.01, **** *p* < 0.0001, ns, not significant.)

Antioxidant properties are of significant importance for tissue regeneration, as they reduce the risk of infection, mitigate inflammatory responses, and establish a favorable microenvironment for tissue repair [44,45]. The free radical scavenging experiments demonstrated that the CMS extract showed merely around 5% scavenging capacity towards DPPH and ABTS radicals at a concentration of 10 mg/mL. Upon incorporation of GC, the antioxidant properties of the hemostatic granules were markedly enhanced. The rate of DPPH and ABTS clearance by CMS-10%GC reached 60%, and further increases in GC loading (CMS-15%GC and CMS-20%GC) resulted in scavenging rates exceeding 80% (Figure 4A–D).

Bacterial infection may occur during the wound healing process, leading to a persistent inflammatory response and delayed wound closure [46,47]. To evaluate the antibacterial efficacy of the hemostatic granules, *Escherichia coli* (*E. coli*) and *Staphylococcus aureus* (*S. aureus*), two common wound pathogens, were selected as model Gram-negative and Gram-positive bacteria. The antibacterial activity of the hemostatic granules was evaluated qualitatively using the spread plate assay. Visual inspection of the agar plates revealed abundant bacterial colonies in the PBS control group and the CMS group. In the CMS-10%GC group, a considerable number of colonies were still observed, suggesting that the relatively low GC content resulted in insufficient bactericidal efficacy. In striking contrast, the CMS-15%GC and CMS-20%GC groups exhibited significantly reduced bacterial colonization, with few colonies observed, indicating potent antibacterial activity (Figure 4E). These qualitative observations were further corroborated by quantitative bactericidal rates determined via CFU enumeration. Against *E. coli*, the CMS-15%GC and CMS-20%GC groups achieved killing rates of 97.12 ± 2.04% and 97.25 ± 1.48%, respectively. Against *S. aureus*, the corresponding antibacterial rates were 86.73 ± 1.27% and 92.03 ± 1.37%, respectively (Figure 4F,G). In parallel, absorbance measurements at 600 nm were performed as a complementary approach to monitor overall bacterial growth trends. The OD600 results demonstrate a trend consistent with the CFU data, revealing a GC concentration-dependent enhancement of antibacterial activity (Figure S15). Furthermore, the antibacterial activity was assessed against ATCC-derived *E. coli*, *S. aureus*, and *methicillin-resistant Staphylococcus aureus* (*MRSA*), a clinically important drug-resistant pathogen. Visual inspection of the agar plates showed that CMS-15%GC and CMS-20%GC displayed good antibacterial activity against all three strains, with *MRSA* being equally susceptible (Figure S16). The antibacterial performance of CMS-15%GC and CMS-20%GC was found to be comparable to that of the commercial hemostatic agent Celox [43]. Pure CMS exhibited negligible antibacterial activity under extract-based testing conditions, indicating that the inorganic framework itself does not contribute significantly to antibacterial effects. With the increase in GC loading, the antibacterial efficacy of the extracts was progressively enhanced, suggesting that the activity is primarily governed by GC-derived soluble components. Therefore, despite GC-induced changes in pore structure, the antibacterial performance is mainly attributed to leachate-mediated biochemical interactions rather than structural factors.

Furthermore, the inhibitory activity against bacterial biofilms was evaluated using crystal violet staining. The biofilms in the control group, CMS group, and CMS-10%GC group were intensely stained. In contrast, the biofilms treated with CMS-15%GC and CMS-20%GC showed weaker staining, demonstrating that the drug-loaded hemostatic granules significantly inhibited bacterial biofilm formation (Figure 4E). The quantitative analysis confirmed that the lowest biofilm biomass was observed in the CMS-15%GC and CMS-20%GC groups (Figure S17). SEM revealed that, after 4 h of cocultivation with the hemostatic granules, the bacterial surfaces in the control group and CMS group were smooth and intact, whereas slight membrane folds were observed in the CMS-10%GC group. However, the bacterial cells in the CMS-15%GC and CMS-20%GC groups exhibited obvious membrane rupture and cytoplasmic leakage, accompanied by the loss of normal cell morphology (Figure 4H).

The effect on bacterial metabolic activity was analyzed by detecting the ATP content of bacteria after cocultivation with the material to further explore the antibacterial mechanism of the hemostatic granules. For *S. aureus*, ATP content in the CMS-15% GC and CMS-20% GC groups was reduced to 15% of that in the control, whereas for *E. coli*, it was reduced to 10% of the control. These results suggest that the hemostatic granules could exert antibacterial effects by inhibiting ATP synthesis and thereby disrupting bacterial metabolic processes (Figure 4I,J).

The potent antibacterial properties of the hemostatic granules can be attributed to two factors: (1) Polyphenols in the GC extract react with bacterial membrane proteins, inducing membrane structural damage and the leakage of the cellular contents [48,49]. (2) Polyphenols in the GC extract can inhibit bacterial glucose metabolism, thereby reducing bacterial ATP production [50].

### 3.5. Wound Healing by CMS-GC Hemostatic Granules in an Infected Full-Thickness Skin Defect Model

**Figure 5:**
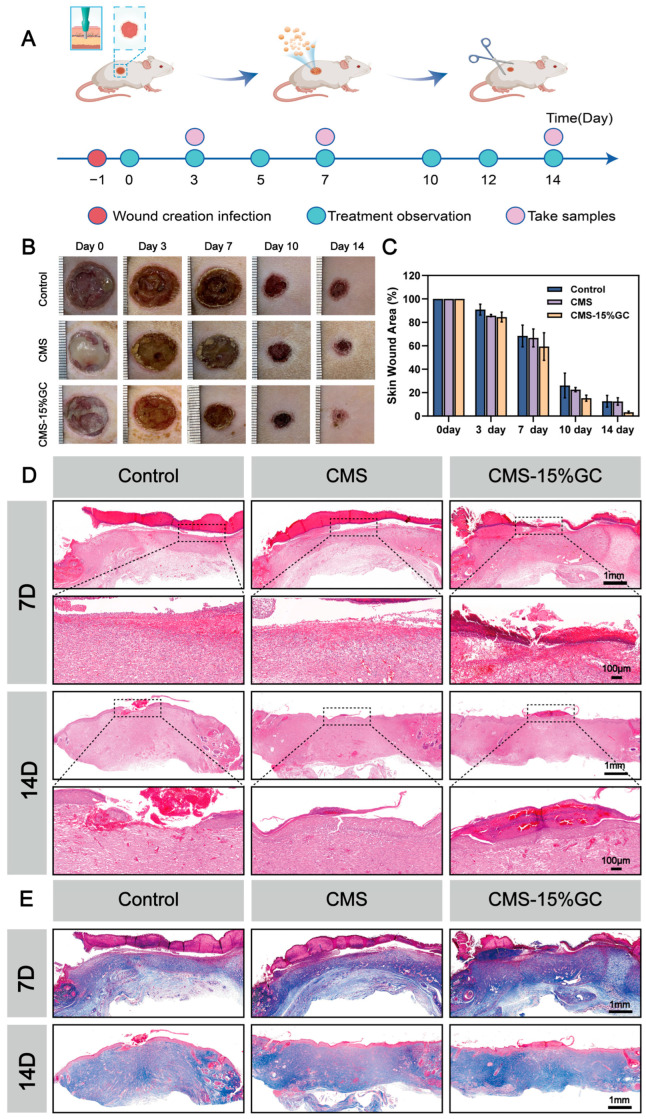
In vivo evaluation of infected wound healing and tissue regeneration. (**A**) Schematic illustration of the experimental design for evaluating the therapeutic efficacy of hemostatic granules in a *Staphylococcus aureus* (*S. aureus*)-infected full-thickness skin wound model. A dorsal full-thickness skin defect was created in mice and inoculated with S. aureus to establish an infected wound model. Animals were randomly assigned to control (no treatment), CMS, and CMS-15%GC groups for topical application of granules. (**B**) Representative digital images of wounds at days 3, 7, 10, and 14 post-treatment, demonstrating wound contraction and re-epithelialization over time. (**C**) Quantitative analysis of residual wound area at each time point. (**D**) Hematoxylin–eosin (H&E) staining of wound sections at days 7 and 14 to assess inflammatory cell infiltration, re-epithelialization, and granulation tissue formation. (**E**) Masson’ s trichrome staining at days 7 and 14 to evaluate collagen deposition and extracellular matrix remodeling, with collagen fibers stained blue (n = 4 rats per group).

Since previous studies have demonstrated that CMS-GC hemostatic granules exhibit excellent hemostatic, antibacterial, and antioxidant properties, we aimed to further explore their potential in promoting wound healing in vivo, particularly under infected conditions. To achieve this, we established a full-thickness skin defect model in S. aureus-infected rats (Figure 5A). The CMS-15%GC formulation was selected as the representative material because in vitro experiments indicated that it had the best performance. Relative to the control group and the non-drug-loaded hemostatic granule group, the wound healing rate of the CMS-15% GC group was substantially accelerated. After treatment, the CMS-15%GC group exhibited the minimal wound area among all groups (Figure 5B,C).

A histological analysis was performed to further assess the quality of wound healing. HE staining (Figure 5D) showed obvious tissue defects in each group on the 7th day, but less inflammatory cell infiltration was observed in the CMS-15%GC group. On the 14th day, the healing rate of the control group was slow, and a continuous epidermis had not formed. However, in both the CMS and the CMS-15%GC groups, re-epithelialization was completed, and more sebaceous glands were observed in the CMS-15%GC, indicating that the CMS-15%GC treatment had the greatest effect on promoting wound healing. Research has demonstrated that, at the stage of wound proliferation and remodeling, increased and consistent collagen deposition can indicate increased wound healing efficiency [51]. Therefore, Masson’s trichrome staining was conducted to assess collagen accumulation associated with the wound-healing process. At 7 days after surgery, the control group and the CMS group showed less collagen deposition than the CMS-15%GC group did. On day 14, collagen fibers in the control group appeared disrupted and irregular, whereas those in the treated groups displayed integrity, continuity, and an orderly arrangement. These results, together with the HE staining results, proved that the drug-loaded hemostatic granules CMS-15%GC effectively promoted wound healing (Figure 5E). It should be noted that the current in vivo antibacterial efficacy was evaluated using a single-species wound infection model and a limited number of animals per group, which may limit the statistical robustness and generalizability of the findings. From a clinical translation perspective, infections caused by drug-resistant strains or polymicrobial communities are often more challenging and clinically relevant. Therefore, future studies will evaluate the antibacterial activity of CMS-15%GC against clinically isolated drug-resistant strains and establish animal models of polymicrobial or drug-resistant infections with increased sample sizes to further substantiate its therapeutic efficacy. These investigations will provide a more comprehensive assessment of the therapeutic potential of CMS-15%GC in complex infectious environments and strengthen its translational relevance.

Histopathological examinations of major organs in SD rats were conducted to evaluate the biosafety profile of the hemostatic granules. No detectable lesions were observed in the heart, liver, spleen, lungs, or kidneys across any treatment group (Figure S18). These findings indicate that the granules induce minimal organ toxicity, further supporting their favorable biological safety.

### 3.6. In Vivo Anti-Inflammatory Effect and Vascularization Induced by CMS-GC Hemostatic Granules

**Figure 6:**
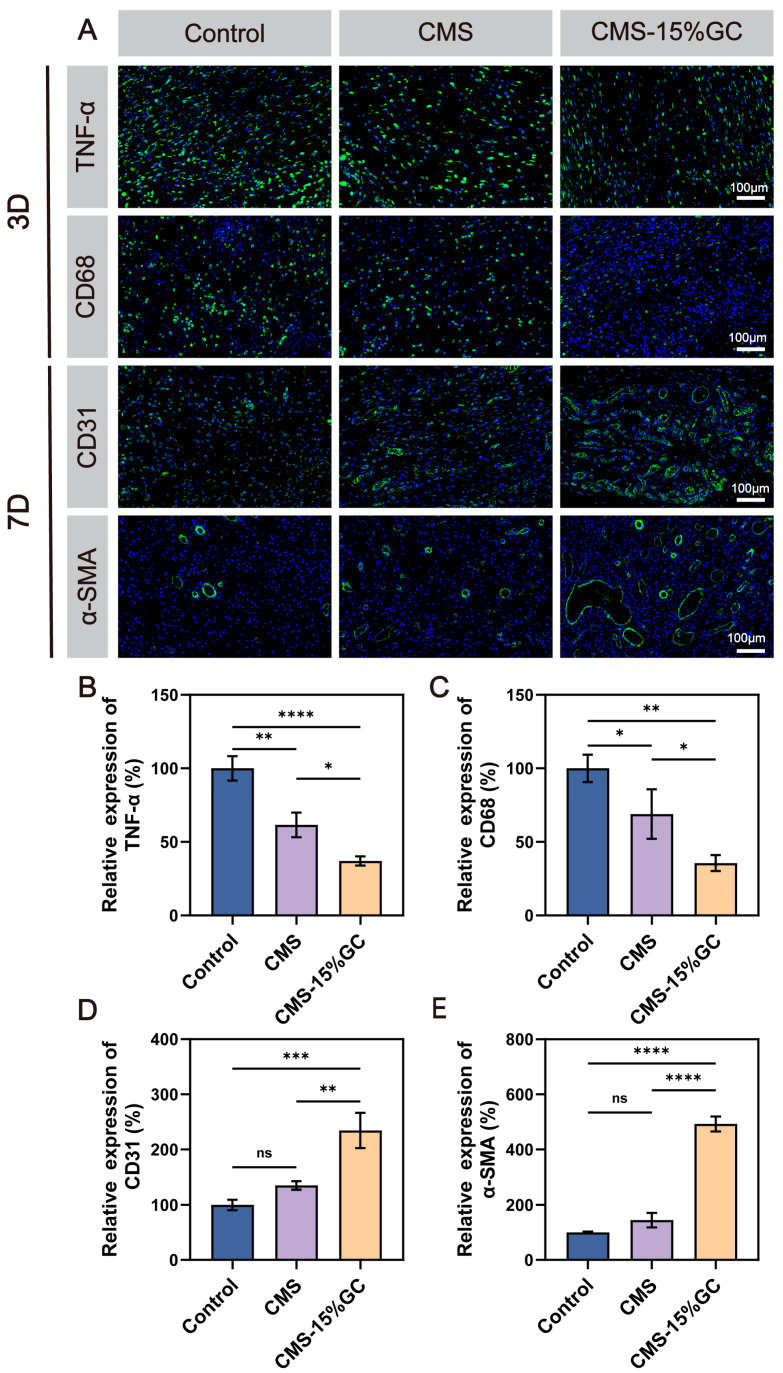
Immunofluorescence analysis of inflammatory response and angiogenesis in infected wounds following CMS-GC treatment. (**A**) Representative immunofluorescence images of wound sections stained for TNF-α and CD68 on day 3 post-treatment to evaluate the early inflammatory response, and for CD31 and α-smooth muscle actin (α-SMA) on day 7 to assess neovascularization and vessel maturation. Green fluorescence indicates TNF-α (pro-inflammatory cytokine), CD68 (pan-macrophage marker), CD31 (endothelial cell marker), and α-SMA (vascular smooth muscle marker). Blue fluorescence corresponds to DAPI-stained nuclei. (**B**) Quantitative analysis of the fraction of TNF-α-positive area. (**C**) Quantitative analysis of the fraction of CD68-positive area. (**D**) Quantitative analysis of CD31-positive micro-vessel density (expressed as the fraction of micro-vessel area). (**E**) Quantitative analysis of the α-SMA-positive vessel maturation area fraction. Data are presented as mean ± standard deviation (SD) (n = 4 rats per group). (* *p* < 0.05, ** *p* < 0.01, *** *p* < 0.001, **** *p* < 0.0001, ns, not significant.)

In the initial repair phase, skin wound healing experiences an inflammatory phase, which significantly affects the quality and speed of healing [52,53]. The inflammatory status of the wound sites on day 3 was assessed across the treatment groups by quantifying tumor necrosis factor-α (TNF-α) levels. On the third day, the expression levels of TNF-α in the control group, CMS group, and CMS-15%GC group decreased from 100% to 61% and 37%, respectively (Figure 6A,B). Additionally, the levels of CD68, a pan-macrophage marker, were measured on the third day to further assess the inflammatory state in the wound area. Among all groups, the CMS-15%GC treatment resulted in the lowest level of macrophage infiltration, aligning well with the observed TNF-α trends (Figure 6A,C). The antibacterial activity of GC likely contributed to this effect by limiting pathogen proliferation, thereby reducing excessive inflammatory stimuli and local tissue damage. Concurrently, the antioxidant properties of CMS-15%GC mitigated ROS accumulation, helping to preserve cellular viability and maintain a favorable environment for subsequent tissue remodeling.

Macrophages play a pivotal role in orchestrating wound healing through dynamic polarization into M1 and M2 phenotypes. The early reduction in inflammation and oxidative stress observed in the CMS-15%GC group may favor a pro-repair macrophage phenotype, which can secrete factors that promote angiogenesis and tissue remodeling. The formation of new blood vessels enhances oxygen and nutrient delivery to the wound site, thereby supporting cell survival, tissue regeneration, and overall wound repair [54]. To evaluate angiogenesis, immunofluorescence staining for CD31 and α-SMA was performed on day 7. Compared with the control and CMS groups, the CMS-15%GC group exhibited more mature vessels (CD31) and higher α-SMA expression, reflecting increased capillary formation and small vessel maturation (Figure 6A,D,E). These findings suggest that CMS-15%GC accelerates wound healing by creating a favorable early wound microenvironment that may promote pro-repair macrophage polarization, mitigating oxidative stress and inflammation, and promoting angiogenesis, collectively contributing to improved tissue regeneration.

## 4. Conclusions

In conclusion, we formulated calcium alginate–mesoporous silica-based hemostatic granules incorporating Galla chinensis extract (CMS-GC) for the management of hemorrhage and wound infections. These granules rapidly adhere to and absorb blood, promote blood cell aggregation to form a robust physical barrier, and further achieve rapid hemostasis at the site of injury. The large specific surface area of CMS ensures uniform distribution of GC within its porous structure. The porous structure of CMS facilitates cross-linking of GC and plasma proteins into a 3D network, thereby accelerating hemostasis. Simultaneously, CMS’s adsorption properties enable sustained GC release, thereby reducing the risk of toxicity. Moreover, the hemostatic granules exhibit excellent biocompatibility, broad-spectrum antimicrobial properties, and antioxidant capabilities. They can eliminate *S. aureus* from infected wounds and diminish oxidative stress at the wound site to modulate inflammation, thereby promoting angiogenesis and accelerating wound repair. In summary, these CMS-GC hemostatic granules integrate four core functions: a rapid hemostatic capacity, antimicrobial effects, antioxidant effects, and immunomodulatory activity. These combined features address the major needs in infected wound care and present a promising therapeutic strategy for clinical wound management.

## Data Availability

The original contributions presented in the study are included in the article, further inquiries can be directed to the corresponding authors.

## Supplementary Materials

The following supporting information can be downloaded at: https://www.mdpi.com/article/10.3390/jfb17060260/s1. Figure S1: Water absorption of CMS granules with different sodium alginate (SA) contents measured at 30 s. CMS-0.025, CMS-0.05, CMS-0.075, and CMS-0.1 represent granules prepared with 0.025, 0.05, 0.075, and 0.1 g of SA, respectively, while the amounts of silicon dioxide (1 g) and calcium carbonate (0.1 g) were fixed; Figure S2: (A) Total ion chromatogram by GC in positive ion mode; (B) Total ion chromatogram by GC in negative ion mode; Table S1: Tentative identification of major chemical components in Galla chinensis extract by LC–MS; Figure S3: Standard curve of Gallic acid; Table S2: Total polyphenols content in GC measured by the Folin-Ciocalteu method; Figure S4: FTIR spectra of MS, SA and CMS granules; Figure S5: FTIR spectra of CMS and CMS-GC granules. (A) O–H and C–H stretching region (~3500–2800 cm^−1^) with C–H peak at 2925 cm^−1^. (B) Fingerprint region (~1800–800 cm^−1^) with COO^−^ vibration at 1378 cm^−1^; Figure S6: Saturated water absorption capacity determined after reaching equilibrium swelling; Table S3: Pore structure characteristics of CMS-GC hemostatic granules; Figure S7: Granule size distribution histogram of CMS and CMS-GC; Figure S8: Drug loading properties of CMS-GC hemostatic particles: (A) Drug loading capacity; (B) Encapsulation efficiency; Figure S9: Thermogravimetric analysis (TGA) curves of CMS and CMS-GC granules; Figure S10: Live/dead staining images following co-culture of L929 cells with different groups of CMS-GC hemostatic granules; Figure S11: In vivo biocompatibility evaluation of CMS-15%GC hemostatic granules: H&E staining of subcutaneous dorsal skin in SD rats at 7 and 14 days post-implantation; Figure S12. Platelet toxicity assay of CMS-GC hemostatic granules; Figure S13: BCI image of CMS-GC hemostatic granules; Figure S14: Calcium-dependent coagulation assay of CMS and CMS-GC; Table S4: Comparison of hemostatic efficacy between CMS-15%GC and commercial Celox in rat hemorrhage models; Figure S15: Quantitative analysis of the bactericidal rate of hemostatic granules determined by OD600 absorbance measurement; Figure S16: Antibacterial activity of CMS-GC hemostatic granules against ATCC-derived *E. coli*, *S. aureus*, and *MRSA*; Figure S17: Quantitative analysis of disrupted *E. coli* and *S. aureus* biofilms following different treatments using crystal violet staining; Figure S18: Histological images of rat heart, liver, spleen, lung and kidney tissues captured 14 days after administration of hemostatic granules.
