# Supplementary material for: Galla Chinensis Polyphenol-Loaded Hemostatic Granules for Rapid Hemostasis, Antibacterial Action, and Wound Healing Promotion
Source: J Funct Biomater. 2026 May 25;17(6):260. doi: 10.3390/jfb17060260 (PMC13302018; doi:10.3390/jfb17060260)
Supplement: Supplementary file 1 [file jfb-17-00260-s001.zip › jfb-4324664-supplementary.pdf]

Supporting information

Galla chinensis polyphenols -loaded hemostatic granules for rapid hemostasis, antibacterial action, and wound healing promotion

*Ruoxue Guo [a, b], Zihan Wu [c], Zirui He [a, b,] *, Changsheng Liu [a, b], Yuan Yuan [a, b,] **

[a] Key Laboratory for Ultrafine Materials of Ministry of Education, School of Materials Science and Engineering, East China University of Science and Technology, Shanghai, 200237, P.R. China

[b] Engineering Research Center for Biomedical Materials of the Ministry of Education, East China University of Science and Technology, Shanghai, 200237, P.R. China

[c] Shanghai Rebone Biomaterials Co., Ltd, Shanghai 201707, P.R. China.

* Corresponding author.

* E-mail address: zirui.he@ecust.edu.cn (Z. He), yyuan@ecust.edu.cn (Y. Yuan).

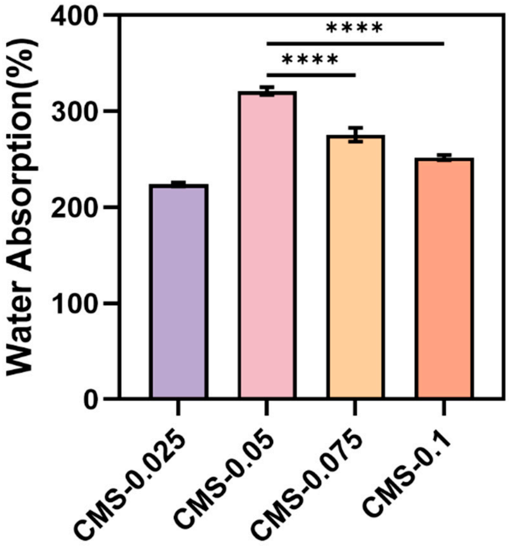

Figure S1. Water absorption of CMS granules with different sodium alginate (SA) contents measured at 30 s. CMS-0.025, CMS-0.05, CMS-0.075, and CMS-0.1 represent granules prepared with 0.025, 0.05, 0.075, and 0.1 g of SA, respectively, while the amounts of silicon dioxide (1 g) and calcium carbonate (0.1 g) were fixed.

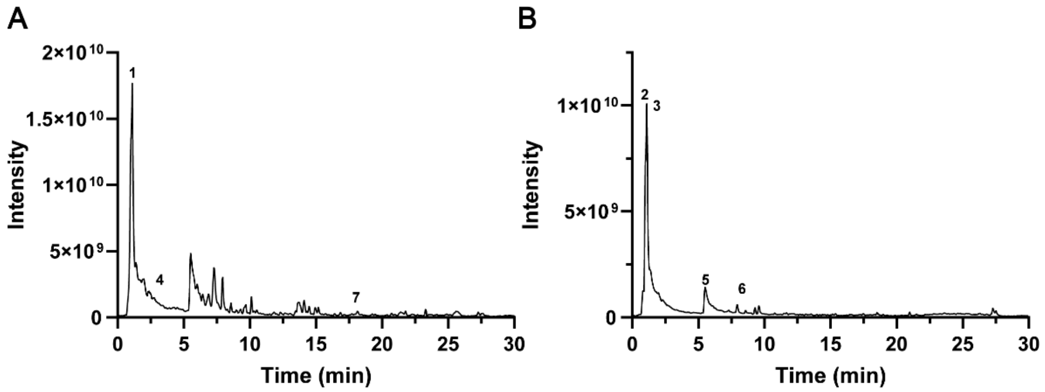

Figure S2. A) Total ion chromatogram by GC in positive ion mode; B) Total ion chromatogram by GC in negative ion mode

Table S1: Tentative identification of major chemical components in Galla chinensis extract by LC–MS.

No.	Identification	Molecular formula	RT (min)	m/z	Adduct ion
1	Pyrogallol	$C_6H_6O_3$	1.06	127.039	$[M+H]^+$
2	Quinic acid	$C_7H_{12}O_6$	1.11	191.0553	$[M-H]^-$
3	4-Hydroxybenzoic acid	$C_7H_6O_3$	1.30	137.0231	$[M-H]^-$
4	Osthole	$C_{15}H_{16}O_3$	2.48	245.1129	$[M+H]^+$
5	Gallic acid	$C_7H_6O_5$	5.54	169.0132	$[M-H]^-$
6	Abscisic acid	$C_{15}H_{20}O_4$	7.30	263.1348	$[M-H]^-$
7	Ellagic acid	$C_{14}H_6O_8$	18.13	303.0063	$[M+H]^+$

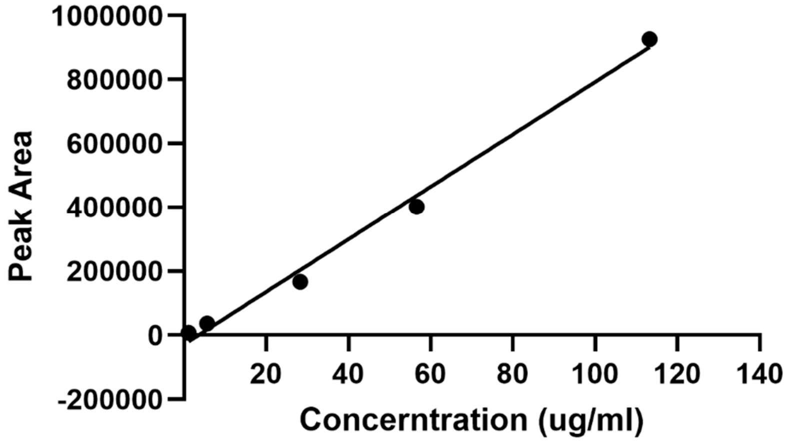

Figure S3. Standard curve of Gallic acid

Table S2: Total polyphenols content in GC measured by the Folin-Ciocalteu method.

Compounds	Concentration (mg/g)
Total polyphenols	89.22 ±5.66

Gallic acid was used as a standard and the results were expressed as mg/g of Gallic acid equivalents.

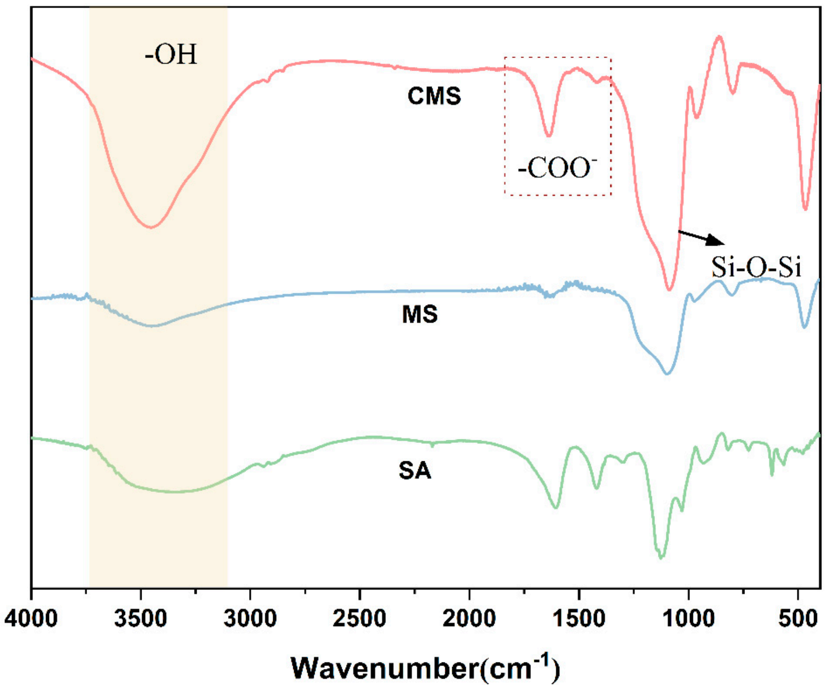

Figure S4. FTIR spectra of MS, SA and CMS granules

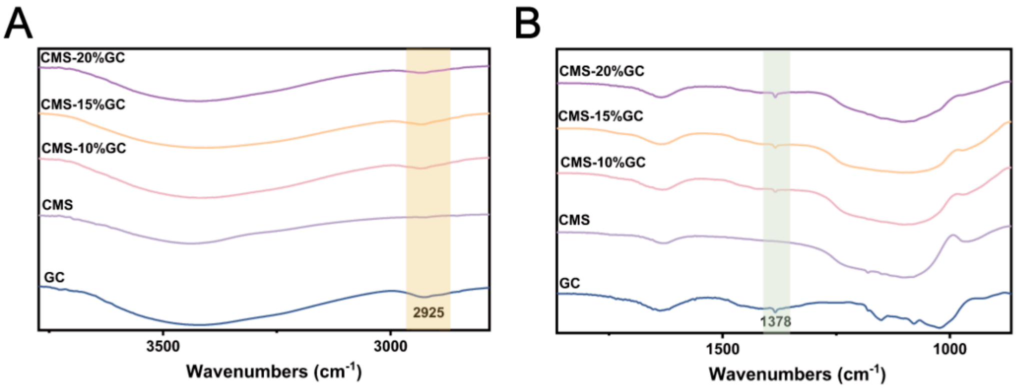

Figure S5. FTIR spectra of CMS and CMS-GC granules. (A) Expanded view of the C–H stretching region around 2925 cm^{-1}. (B) Expanded view of the region around 1378 cm^{-1}, showing coupling of O–H bending, C–H bending, and ring stretching vibrations.

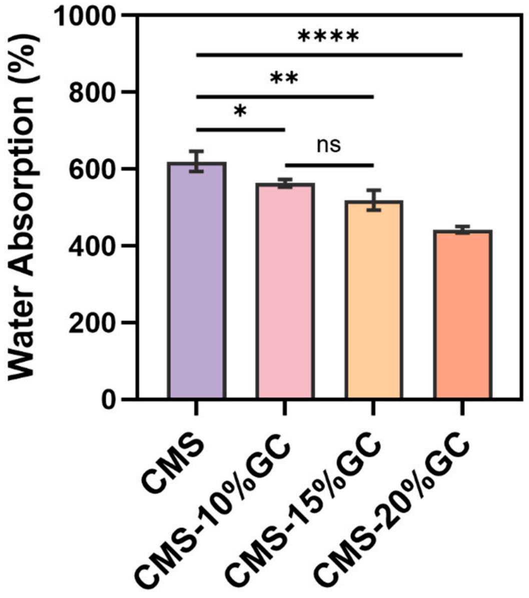

Figure S6. Saturated water absorption capacity determined after reaching equilibrium swelling.

Table S3 Pore structure characteristics of CMS-GC hemostatic granules

	S_{BET} (m^2/g)	V_P (cm^3/g)	D_{BJH} (nm)
**CMS**	509.13	1.47	10.45
**CMS-10%GC**	322.64	1.03	10.91
**CMS-15%GC**	291.09	0.94	10.78
**CMS-20%GC**	216.57	0.72	11.04

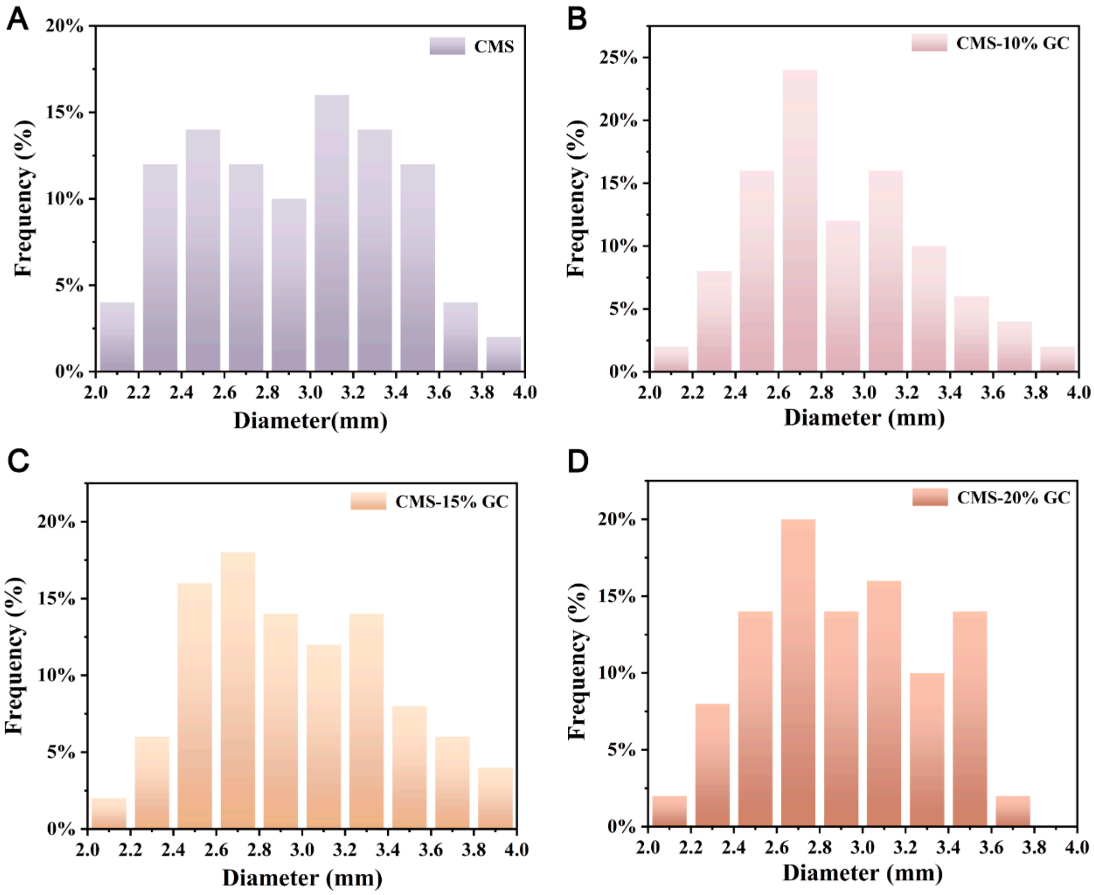

Figure S7. Granule size distribution histogram of CMS and CMS-GC.

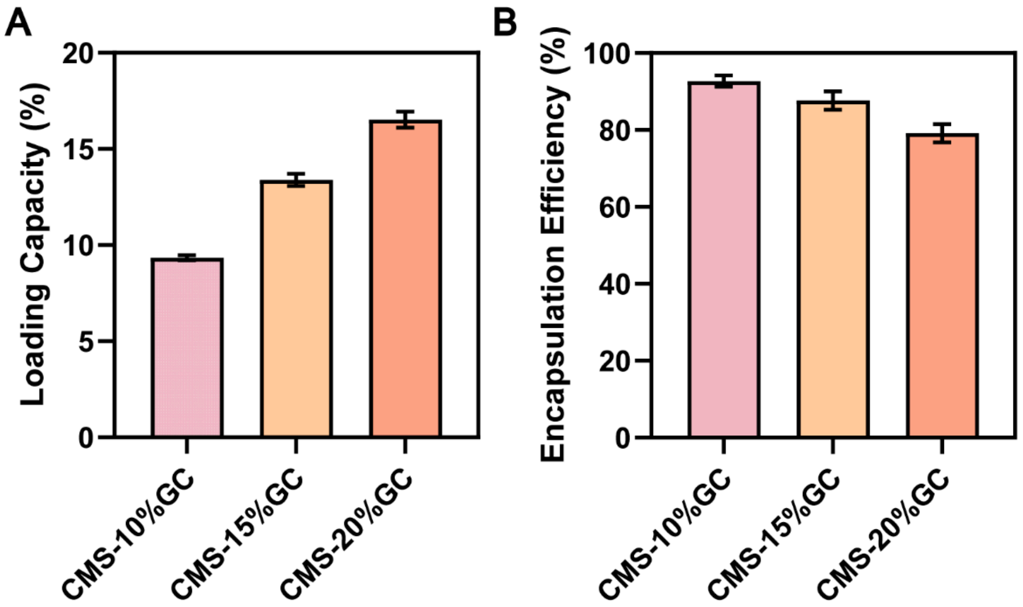

Figure S8. Drug loading properties of CMS-GC hemostatic particles: A) Drug loading capacity; B) Encapsulation efficiency.

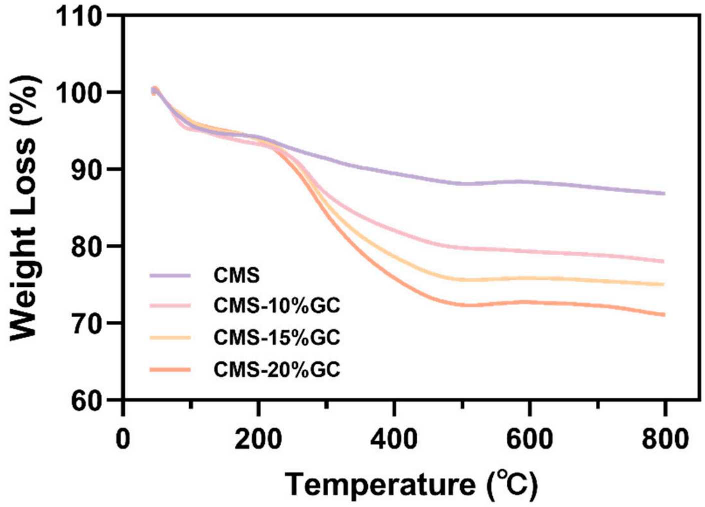

Figure S9. Thermogravimetric analysis (TGA) curves of CMS and CMS-GC granules

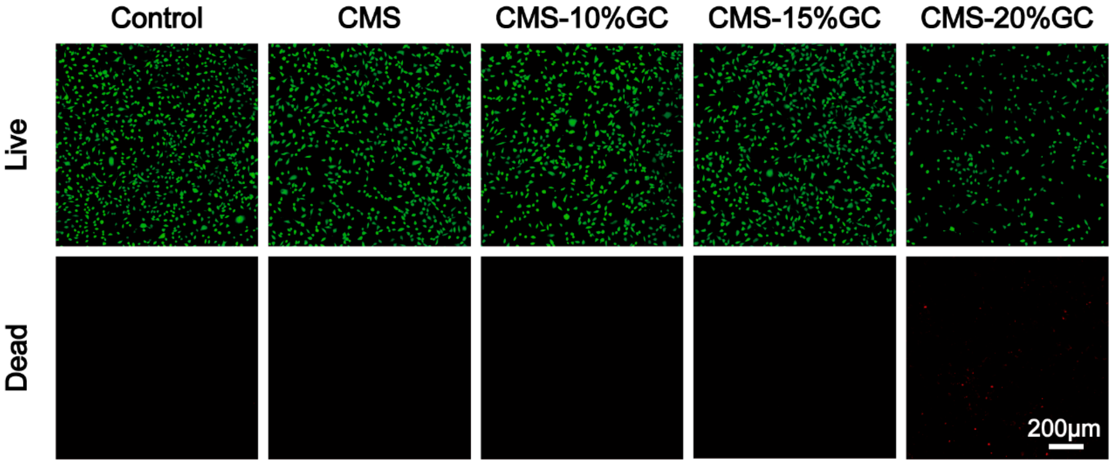

Figure S10. Live/dead staining images following co-culture of L929 cells with different groups of CMS-GC hemostatic granules

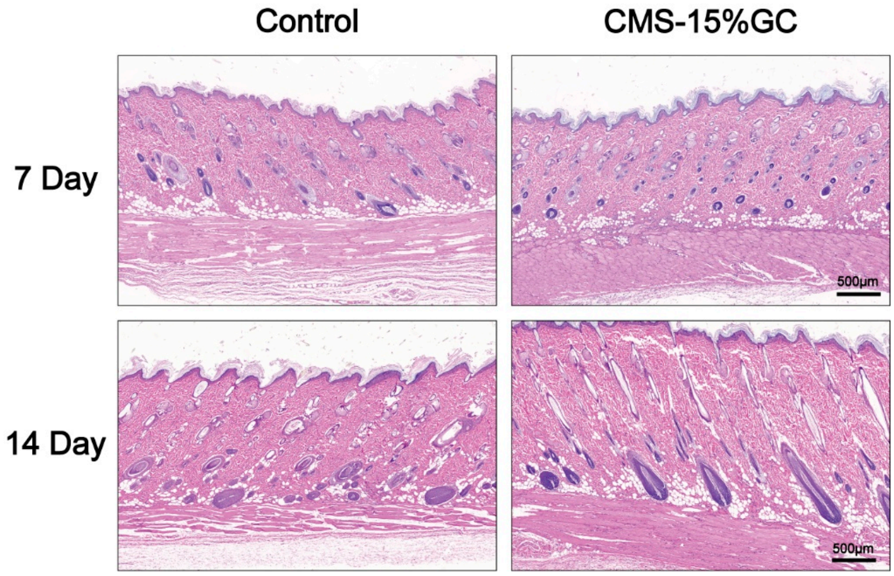

Figure S11. In vivo biocompatibility evaluation of CMS-15%GC hemostatic granules: H&E staining of subcutaneous dorsal skin in SD rats at 7 and 14 days post-implantation.

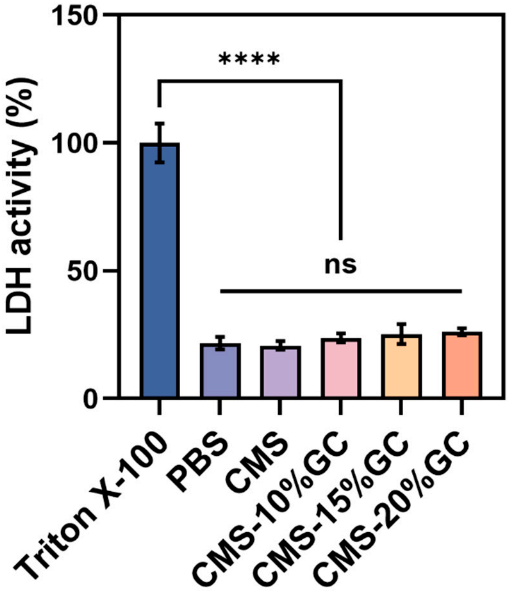

Figure S12. Platelet toxicity assay of CMS-GC hemostatic granules.

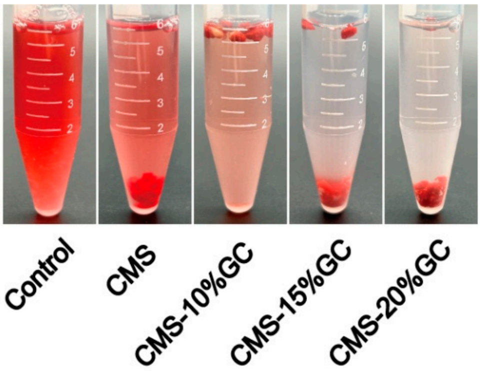

Figure S13. BCI image of CMS-GC hemostatic granules

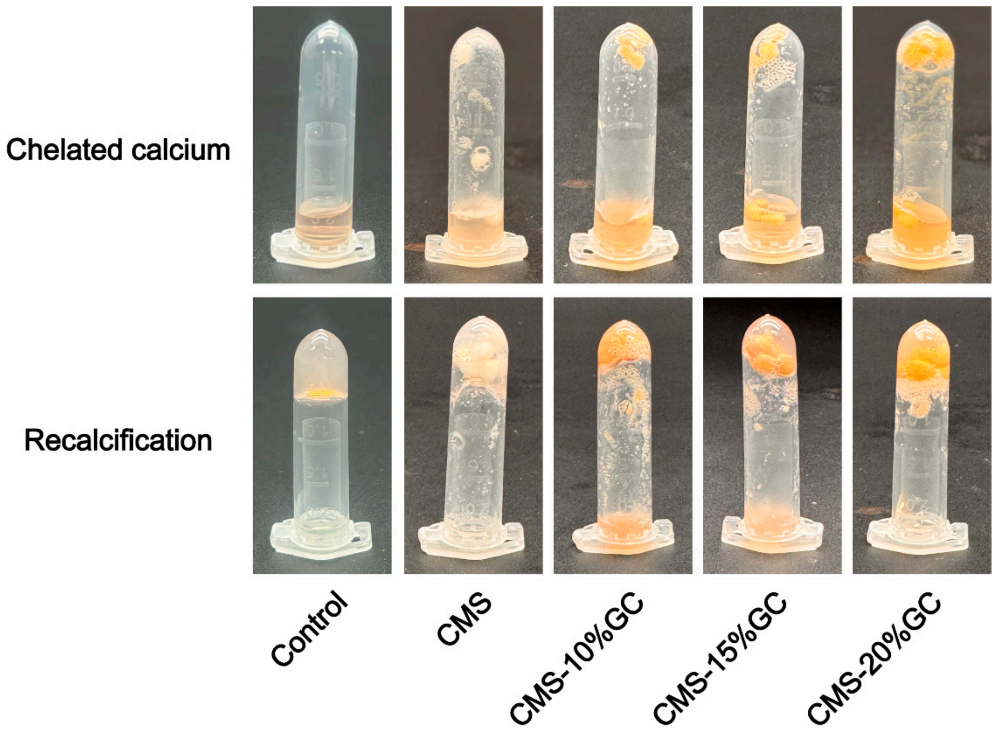

Figure S14. Calcium-dependent coagulation assay of CMS and CMS-GC

Table S4: Comparison of hemostatic efficacy between CMS-15%GC and commercial Celox in rat hemorrhage models.

Model	Parameter	CMS-15%GC	Celox
**Liver hemorrhage**	Hemostasis time (s)	31 ± 3	95 ± 13
	Blood loss (mg)	88 ± 6	348 ± 83
**Femoral artery hemorrhage**	Hemostasis time (s)	93 ± 8	150±16
	Blood loss (mg)	403 ± 20	1074 ± 155

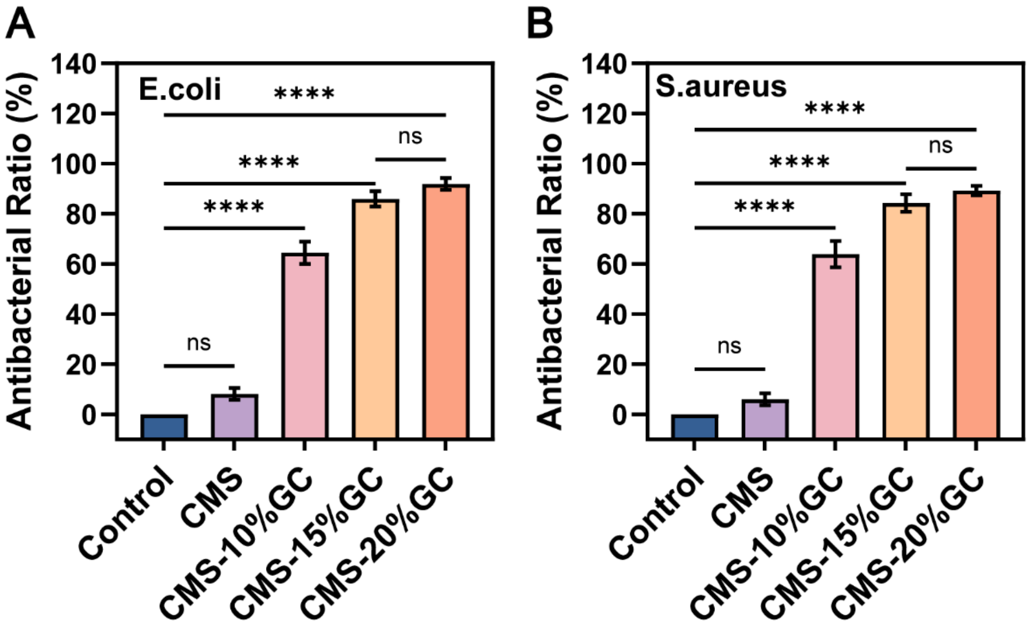

Figure S15. Quantitative analysis of the bactericidal rate of hemostatic granules determined by OD600 absorbance measurement.

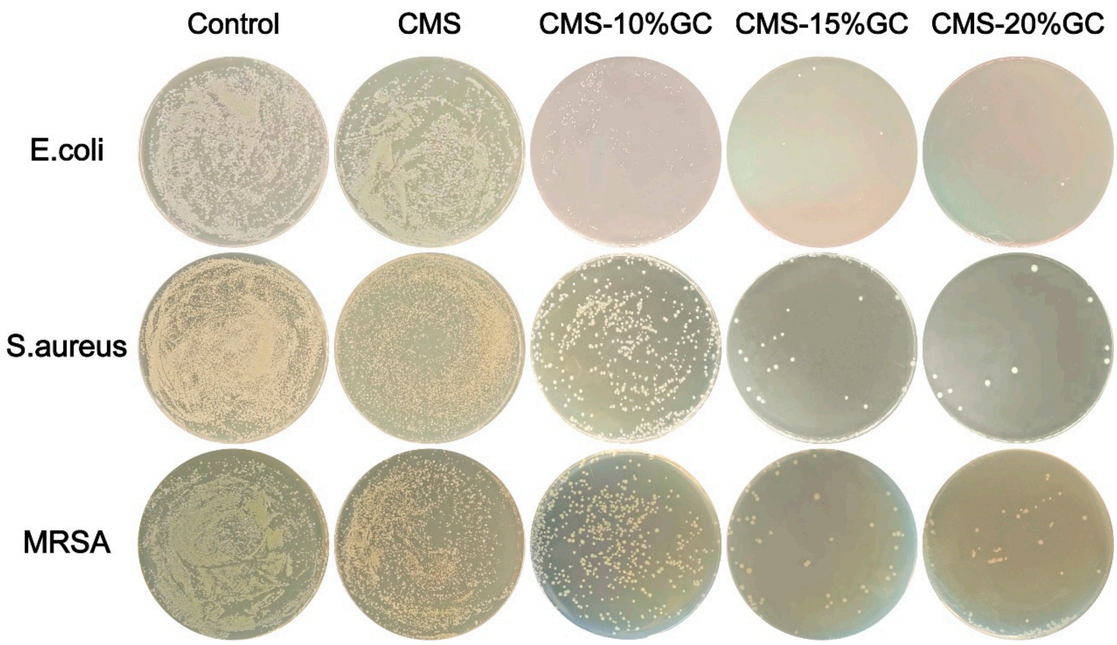

Figure S16. Antibacterial activity of CMS‑GC hemostatic granules against ATCC-derived *E. coli*, *S. aureus*, and *MRSA*.

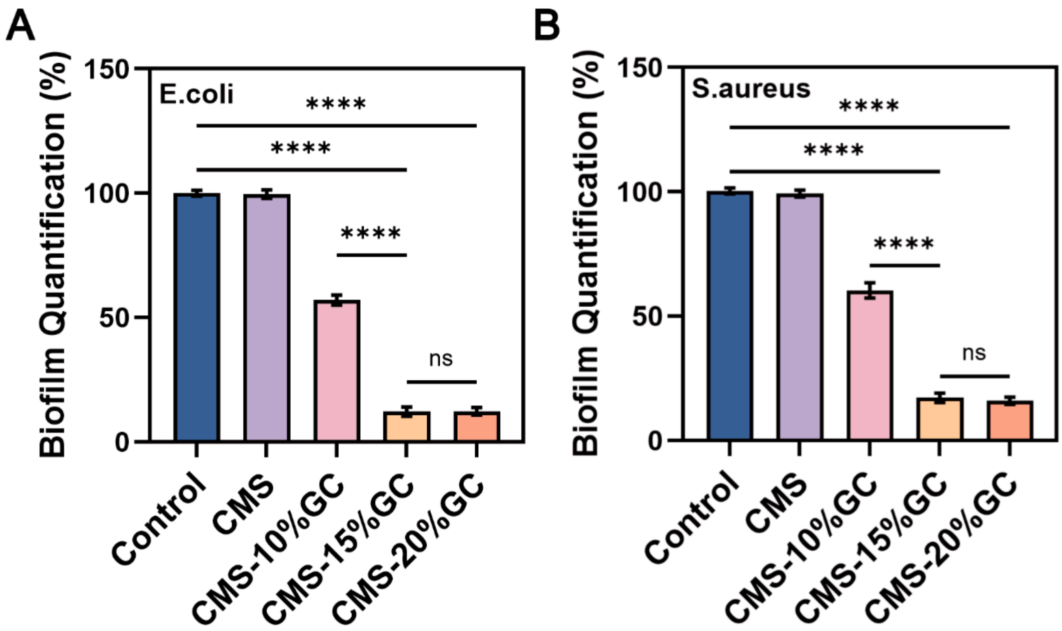

Figure S17. Quantitative analysis of disrupted *E. coli* and *S. aureus* biofilms following different treatments using crystal violet staining

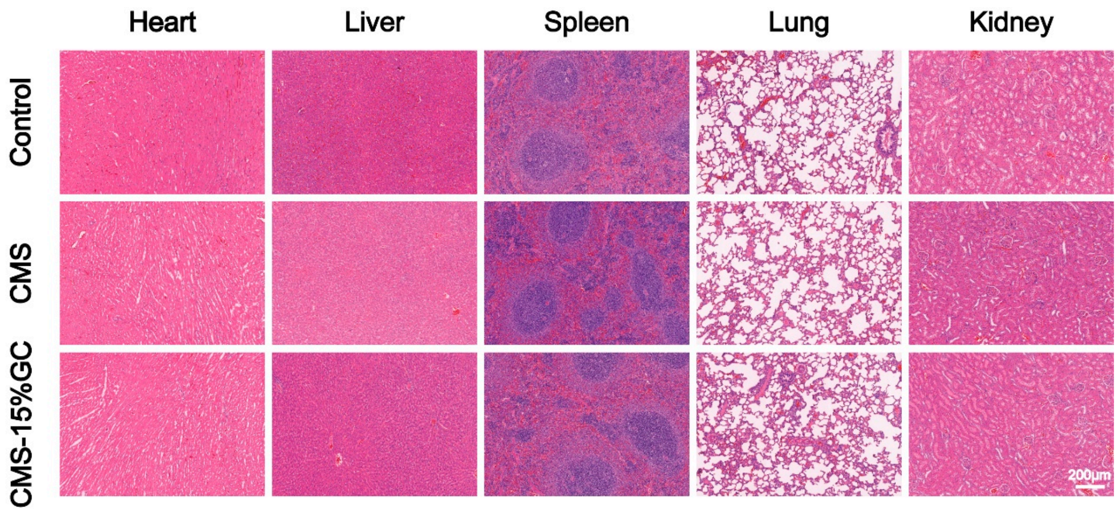

Figure S18. Histological images of rat heart, liver, spleen, lung and kidney tissues captured 14 days after administration of hemostatic granules
